# Supplementary material for: NSUN2 facilitates DICER cleavage of DNA damage-associated R-loops to promote repair
Source: Nat Commun. 2025 Aug 23;16:7882. doi: 10.1038/s41467-025-63220-9 (PMC12374970; doi:10.1038/s41467-025-63220-9)
Supplement: Supplementary file 1 — Supplementary Information [file 41467_2025_63220_MOESM1_ESM.pdf]

Supplementary Fig. 1

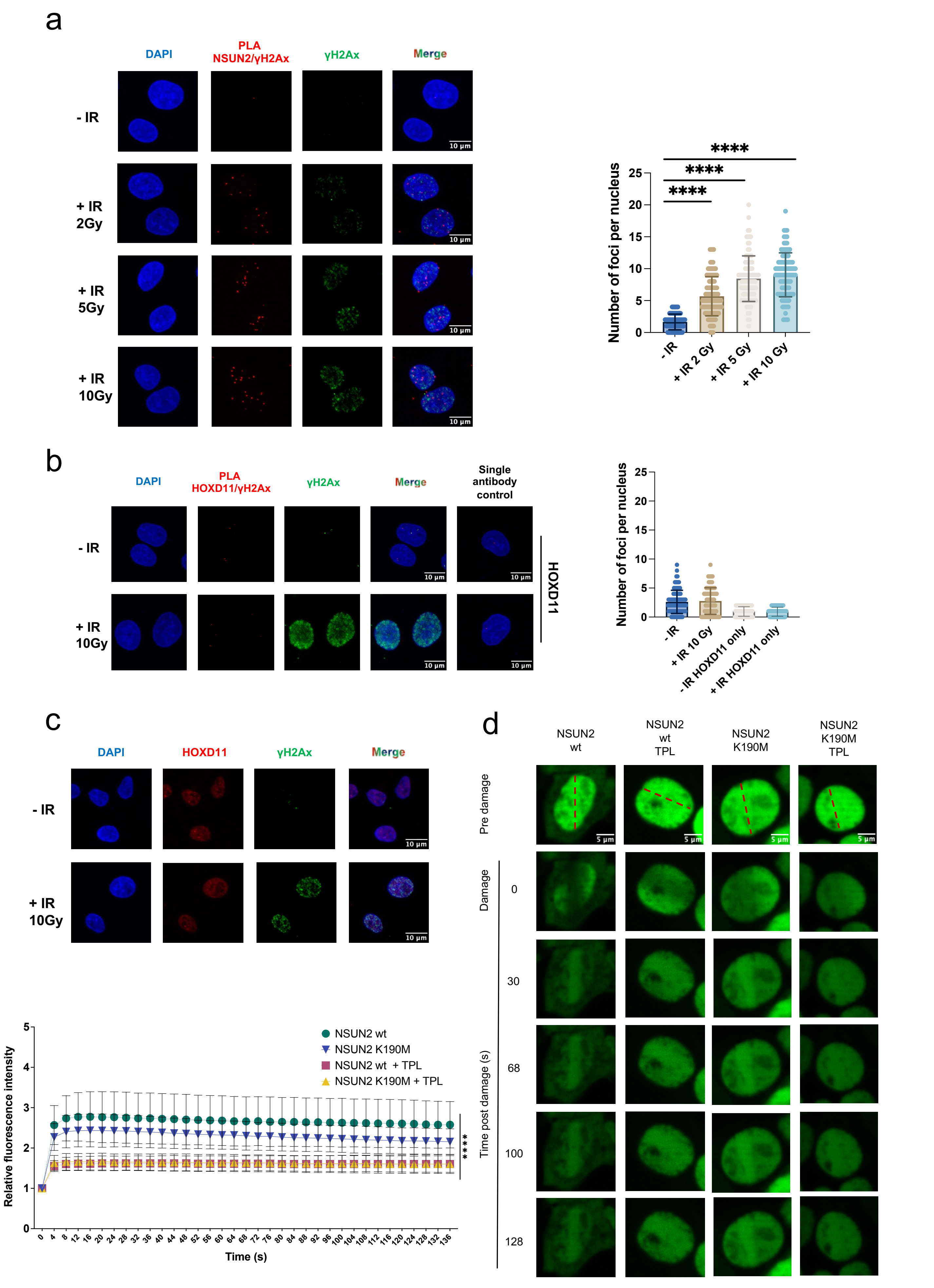

Supplementary Fig. 2

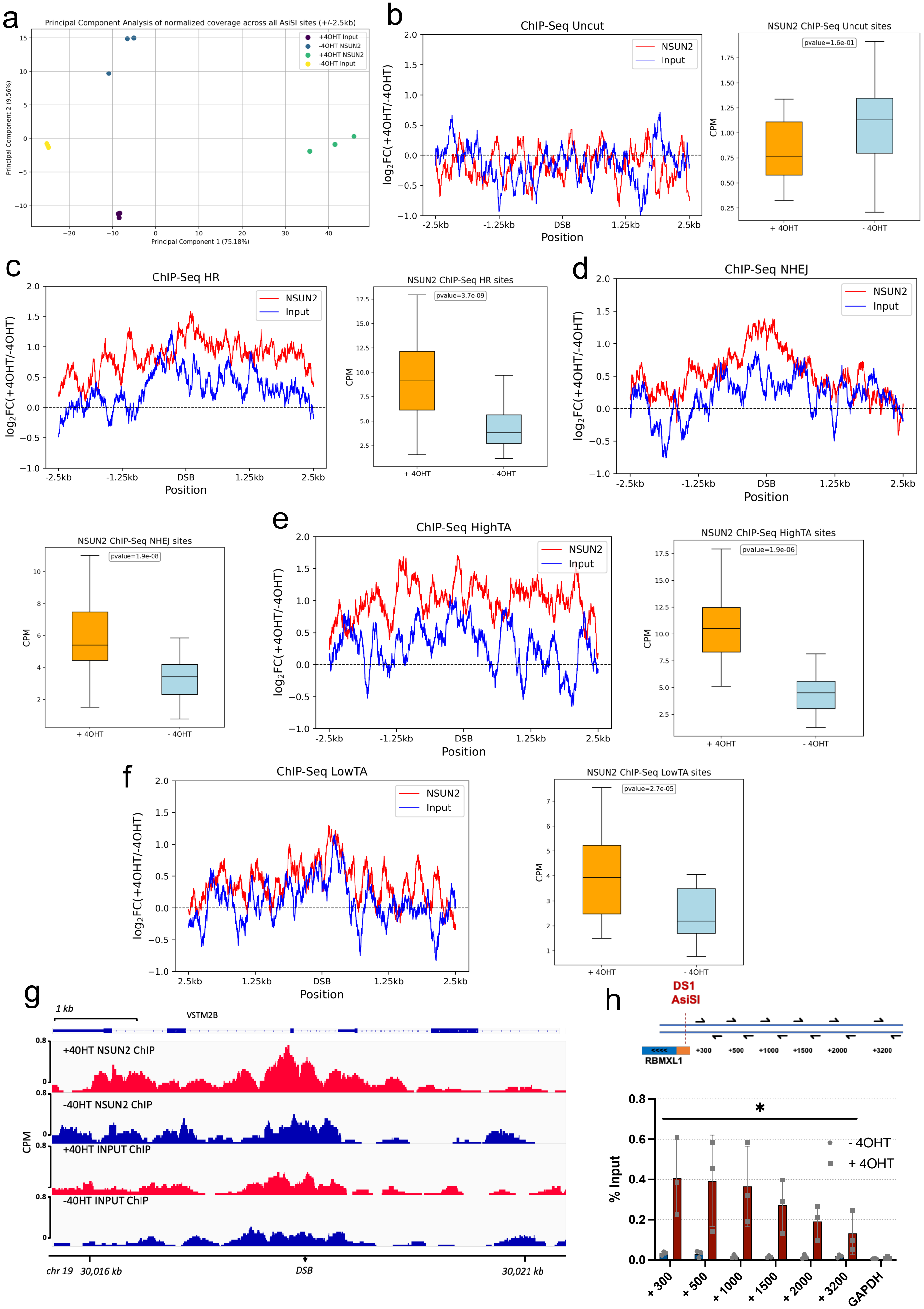

Supplementary Fig. 3

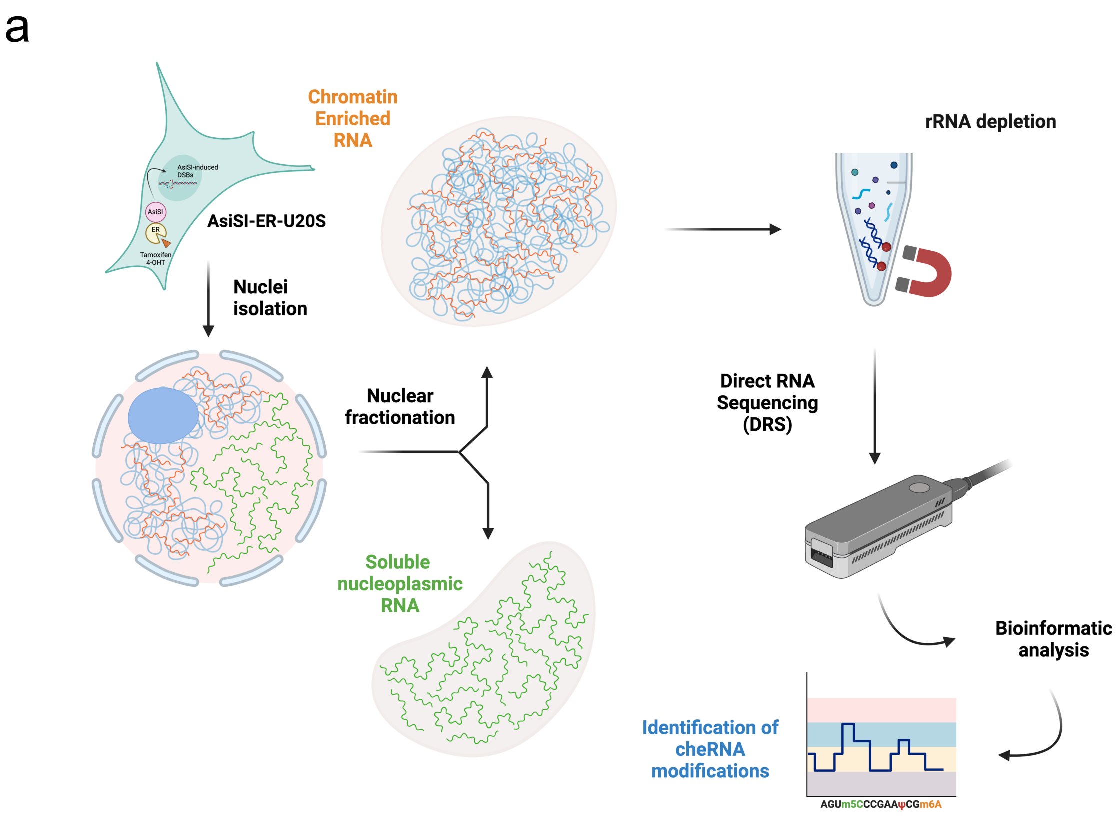

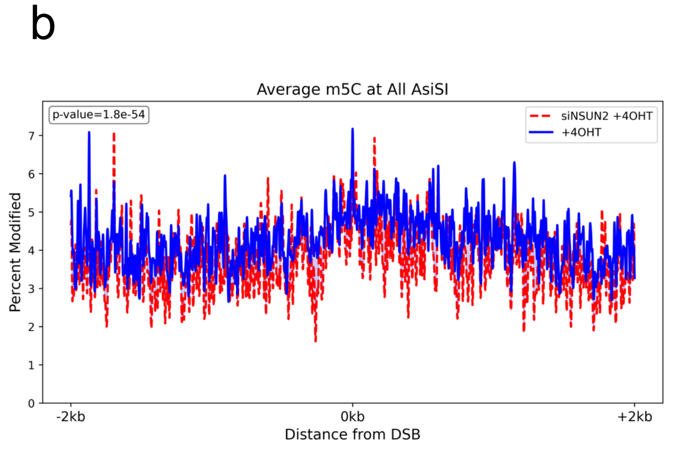

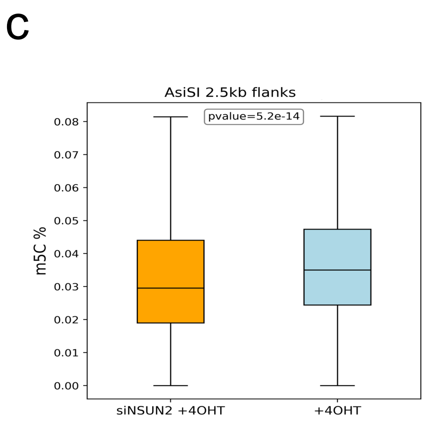

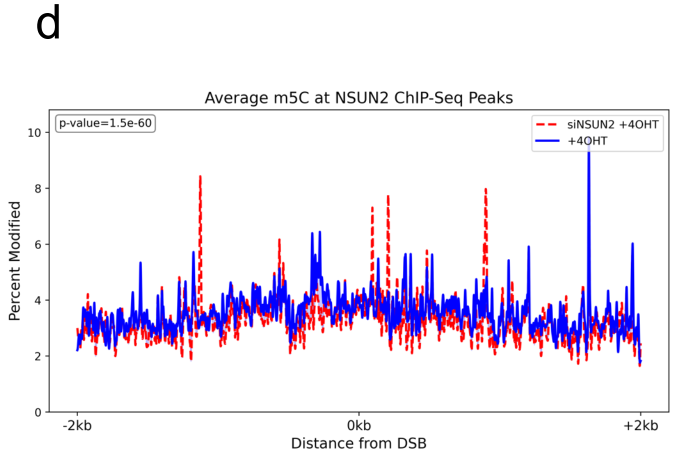

Supplementary Fig. 4

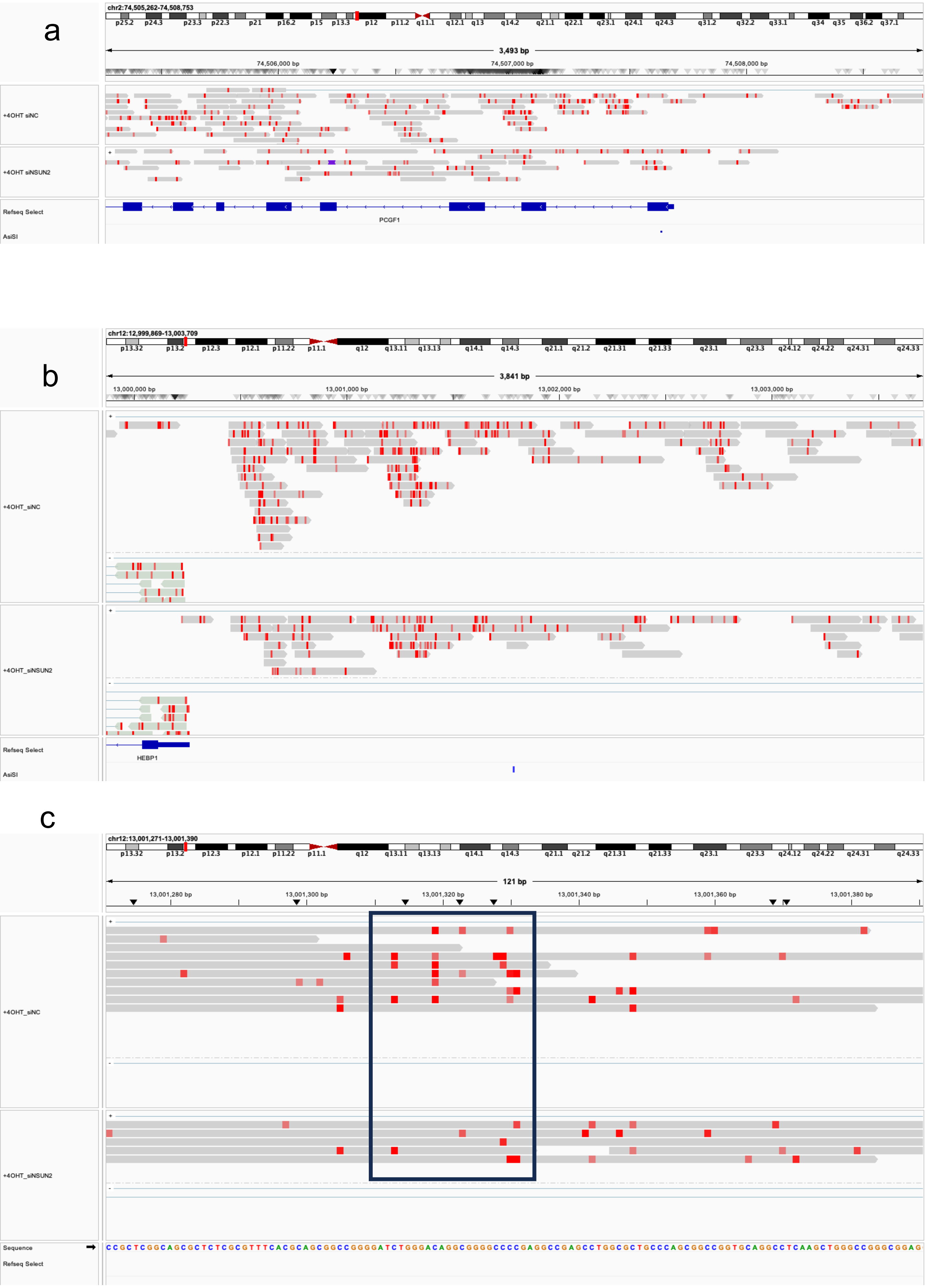

Supplementary Fig. 5

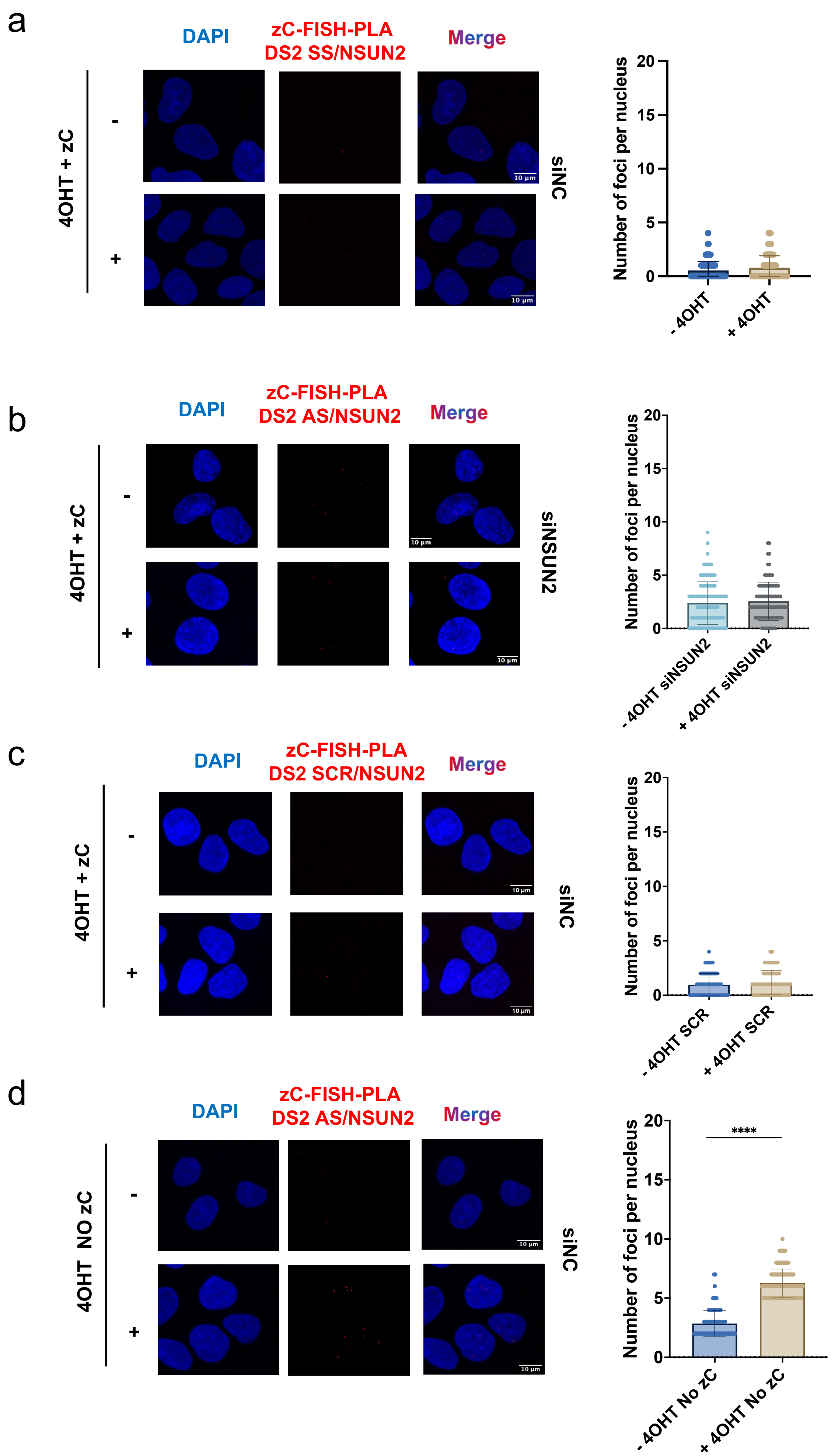

Supplementary Fig. 6

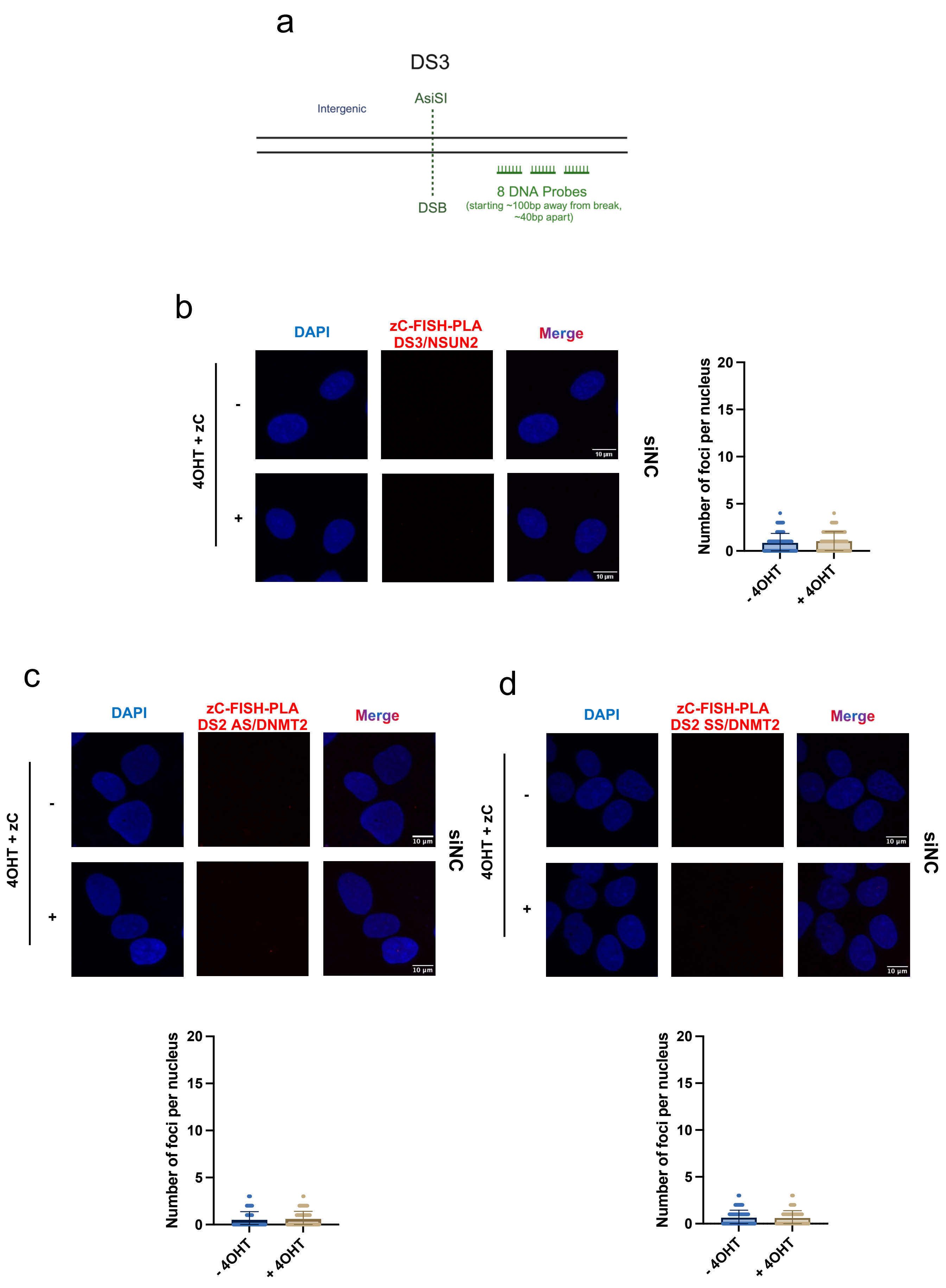

Supplementary Fig. 7

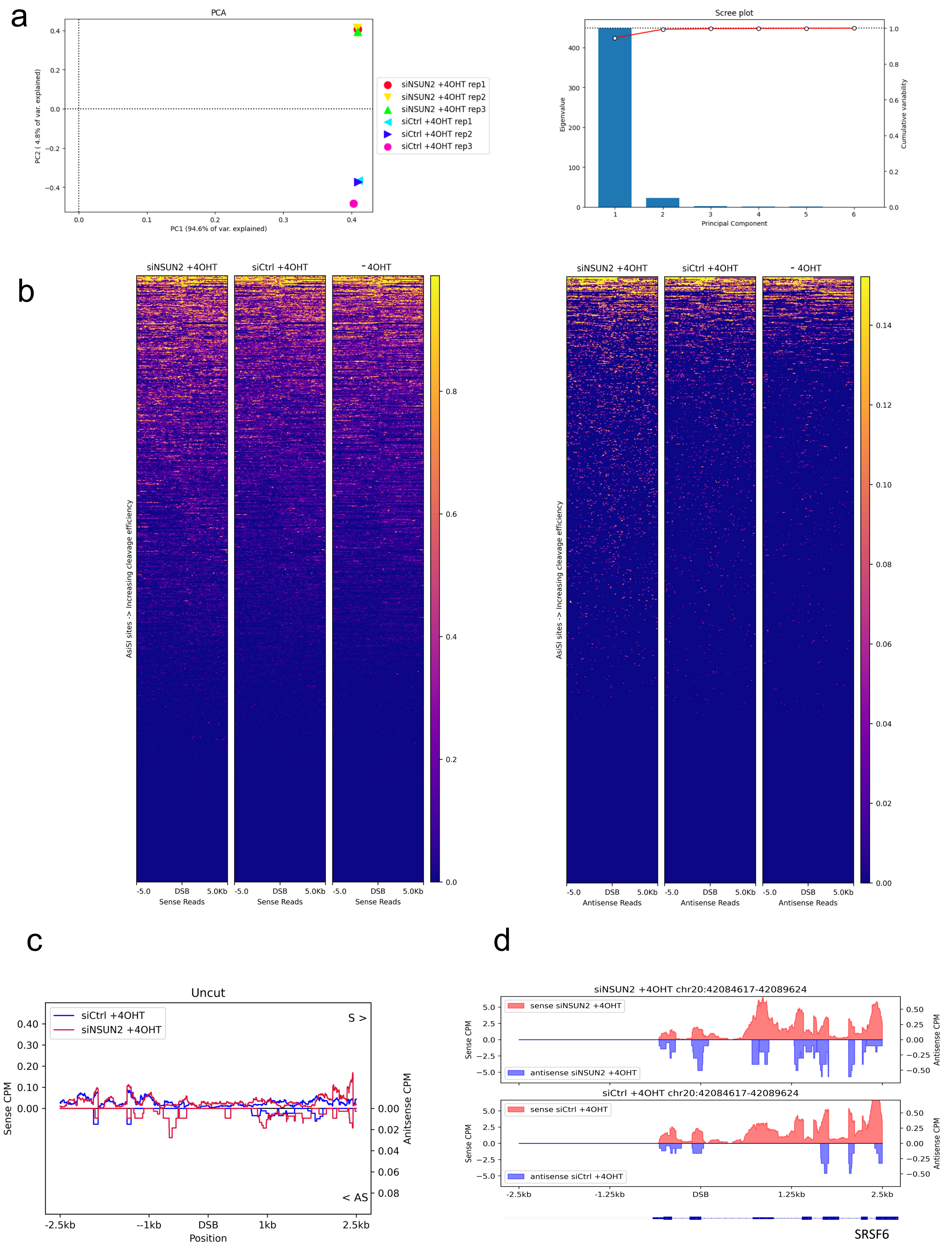

Supplementary Fig. 8

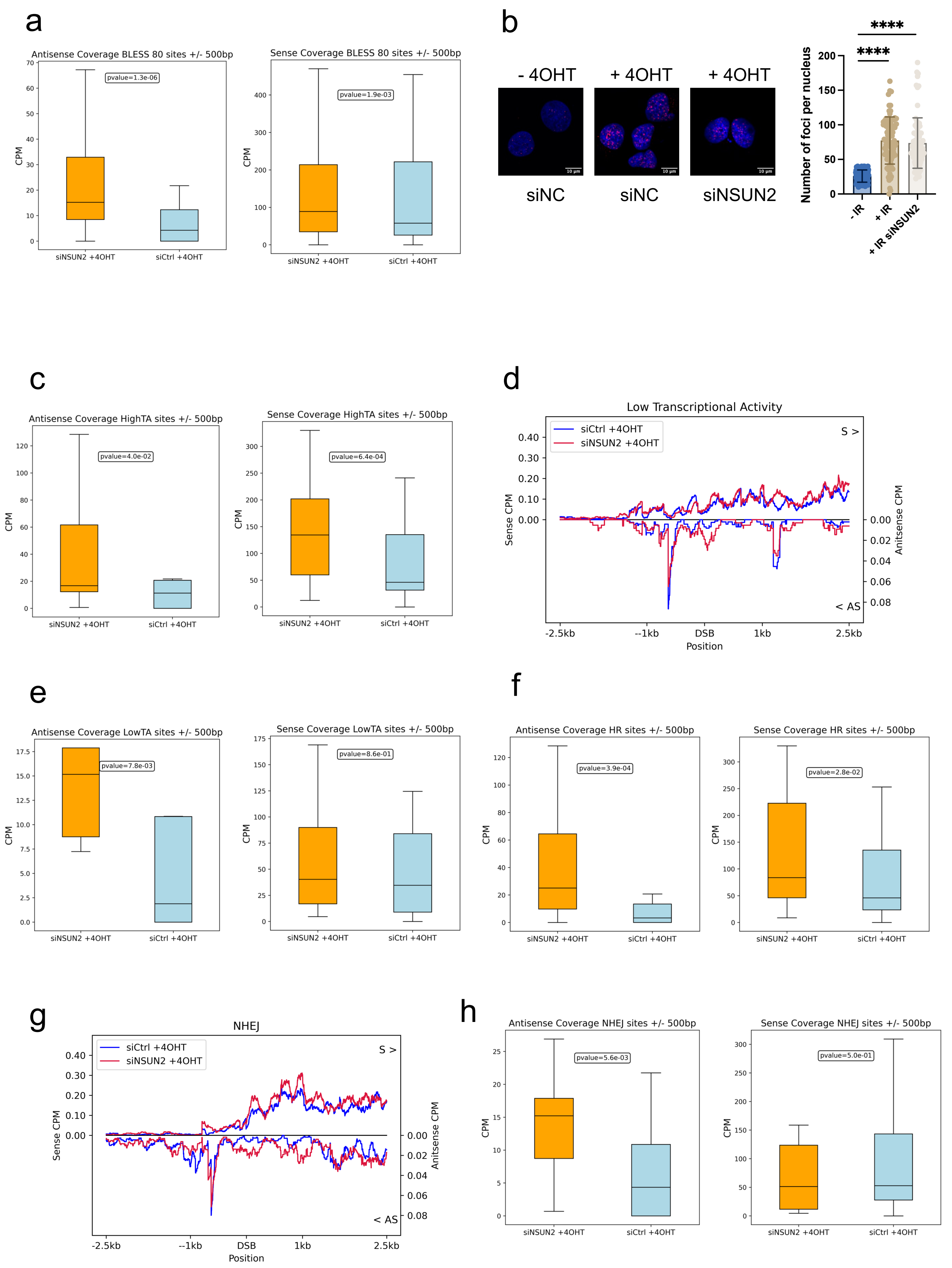

Supplementary Fig. 9

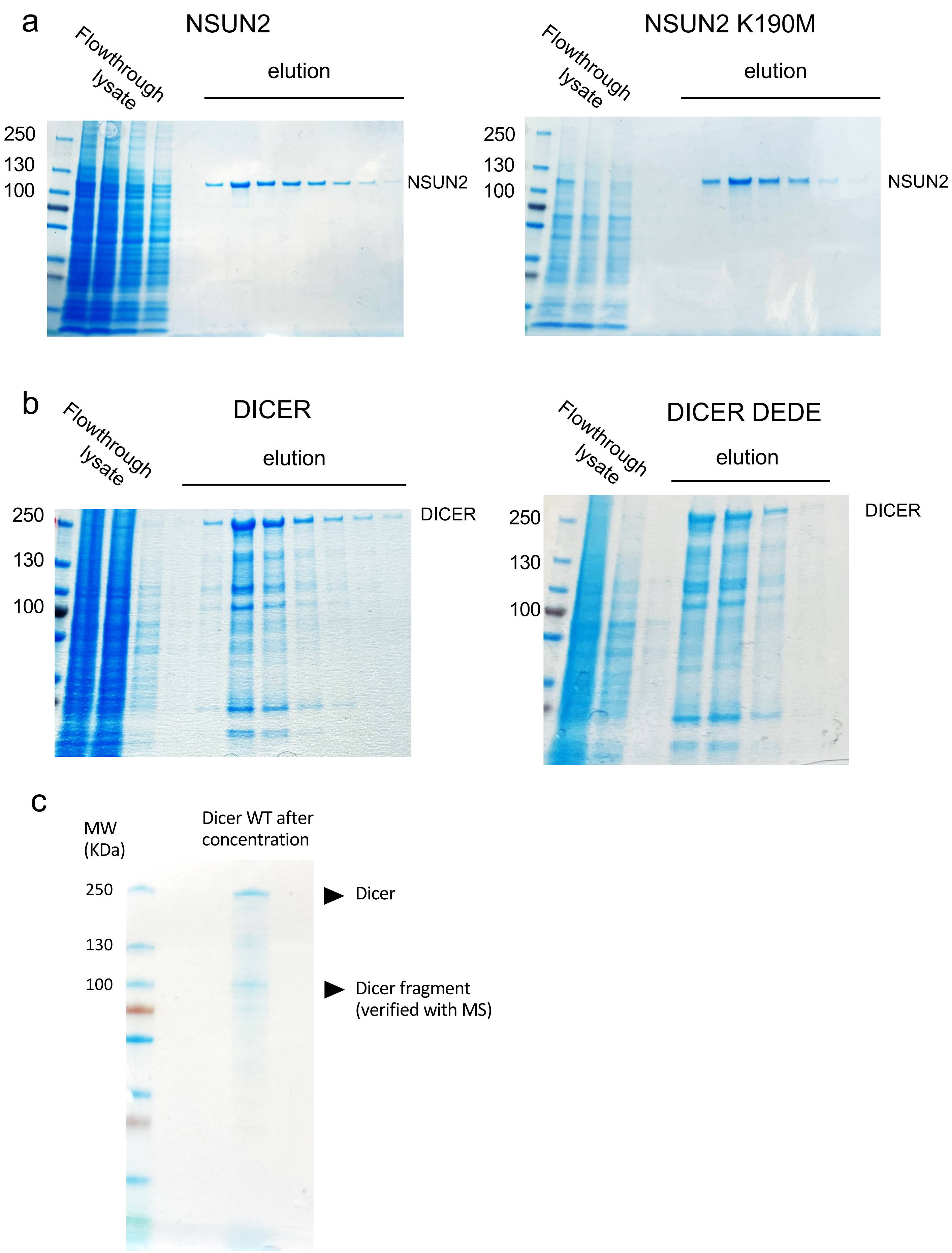

Supplementary Fig. 10

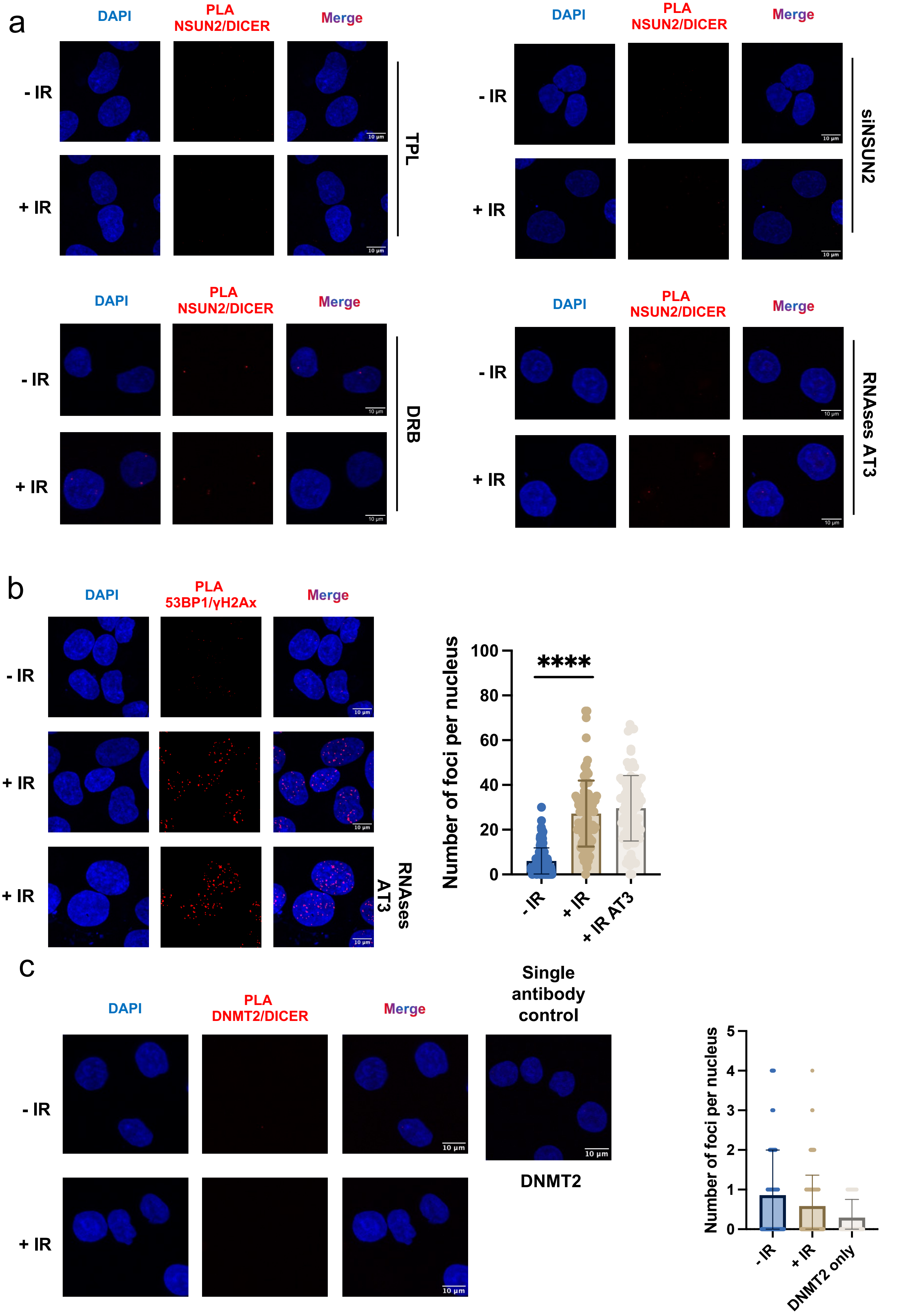

Supplementary Fig. 11

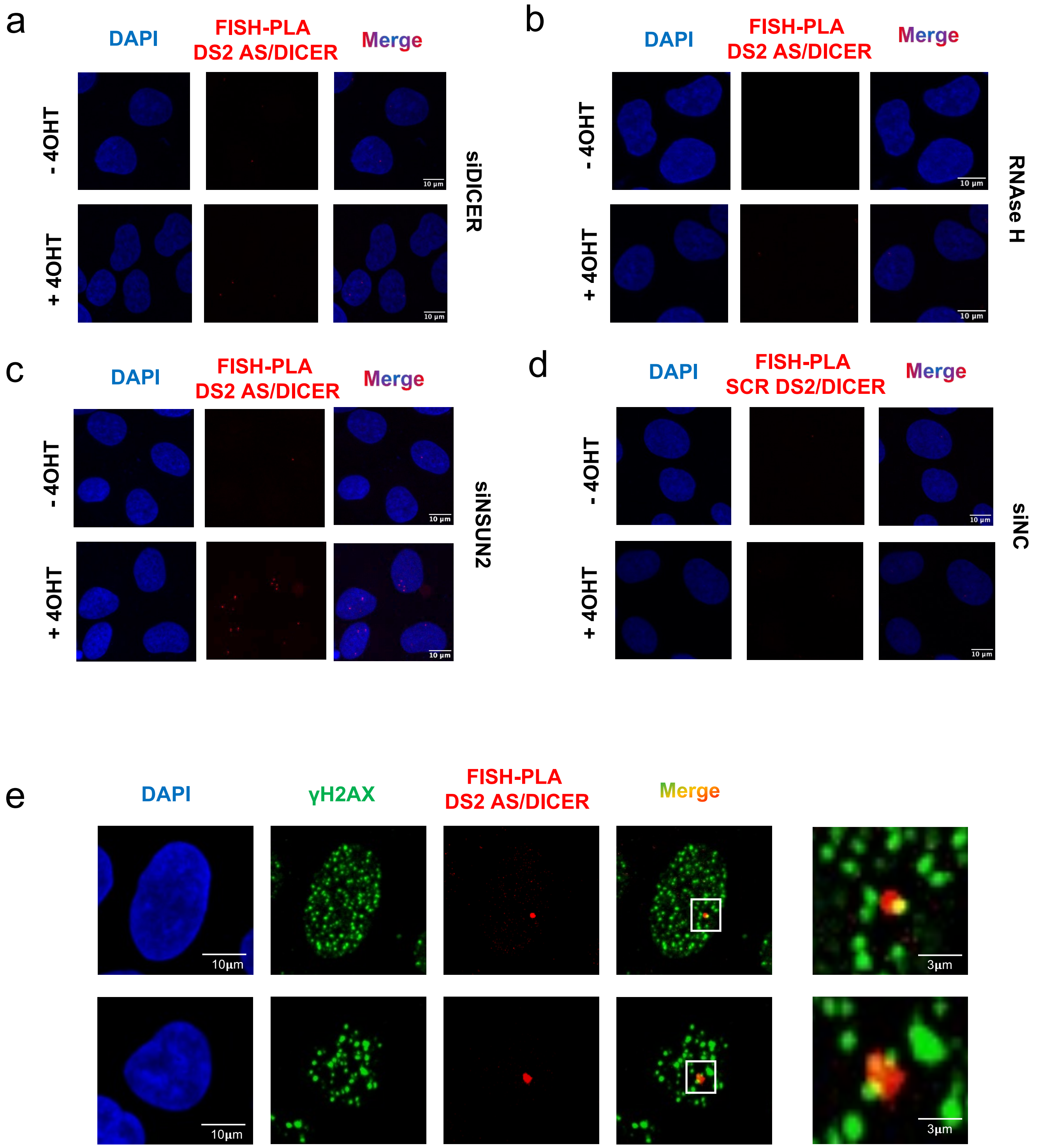

Supplementary Fig. 12

a

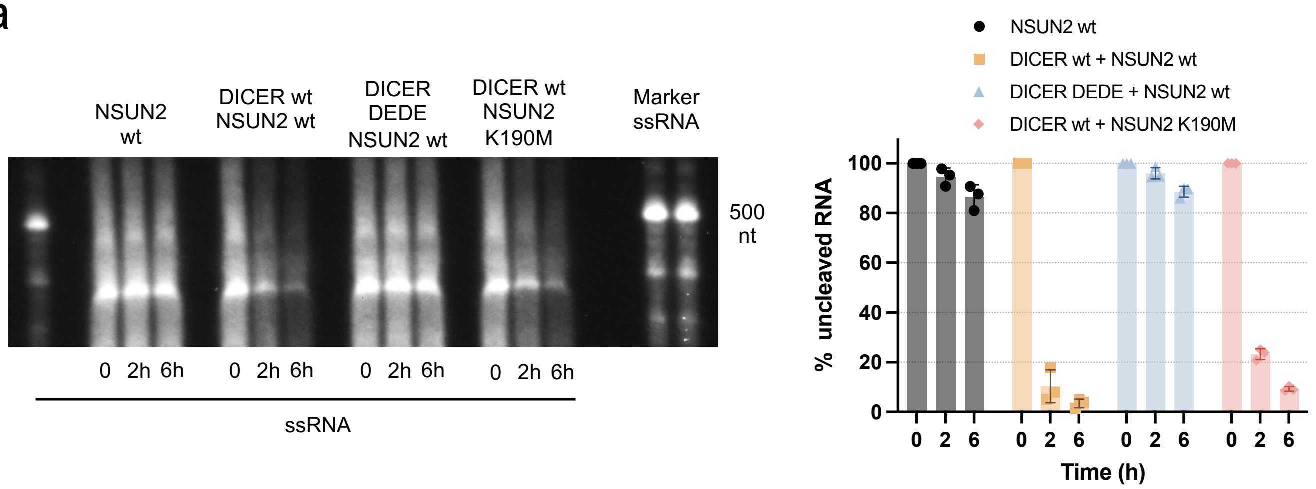

b

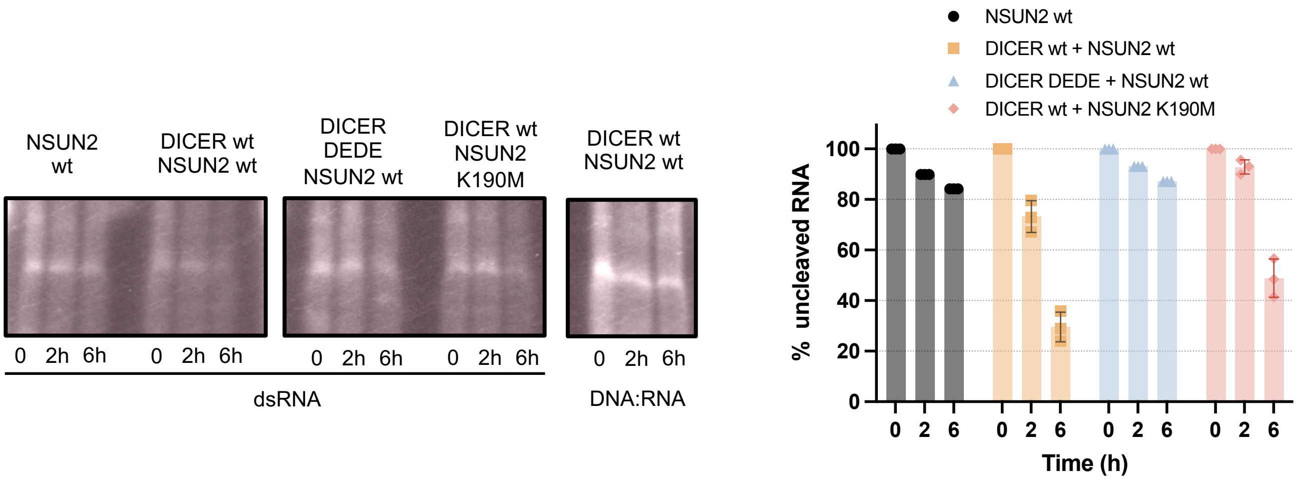

Supplementary Fig. 13

a

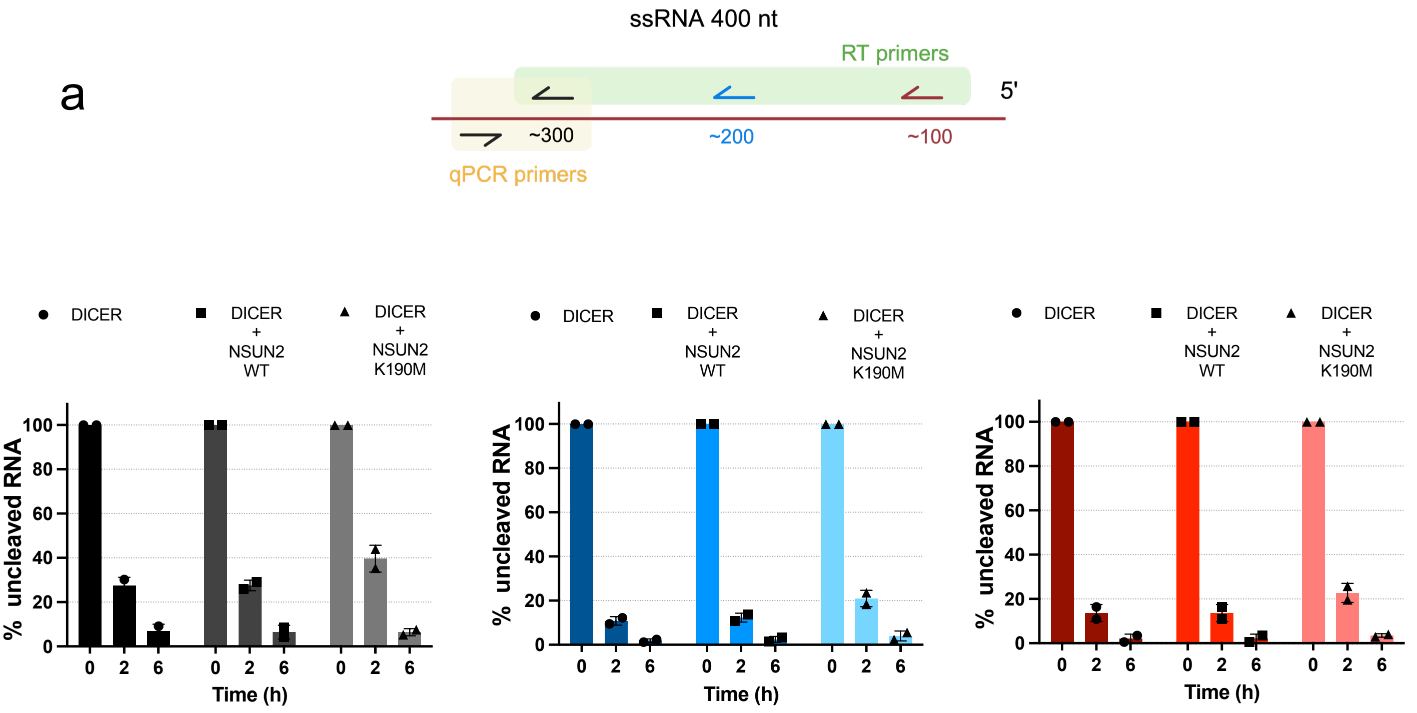

b

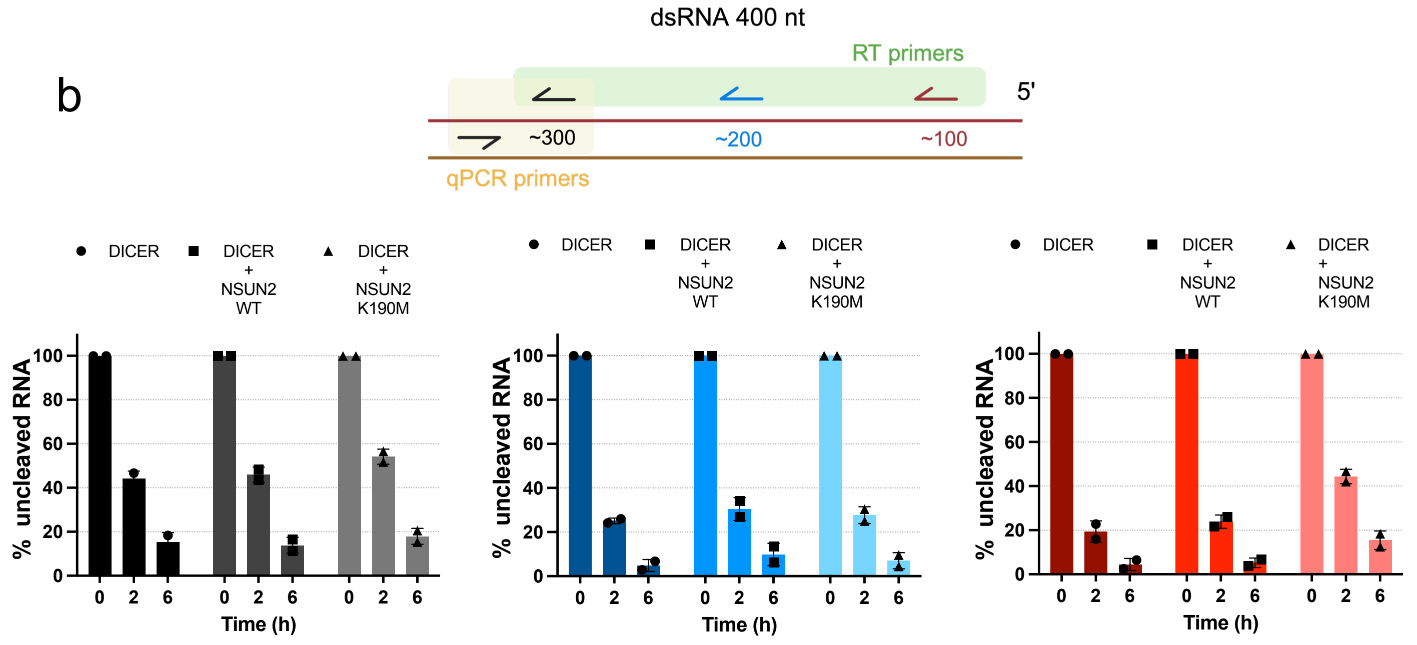

Supplementary Fig. 14

a

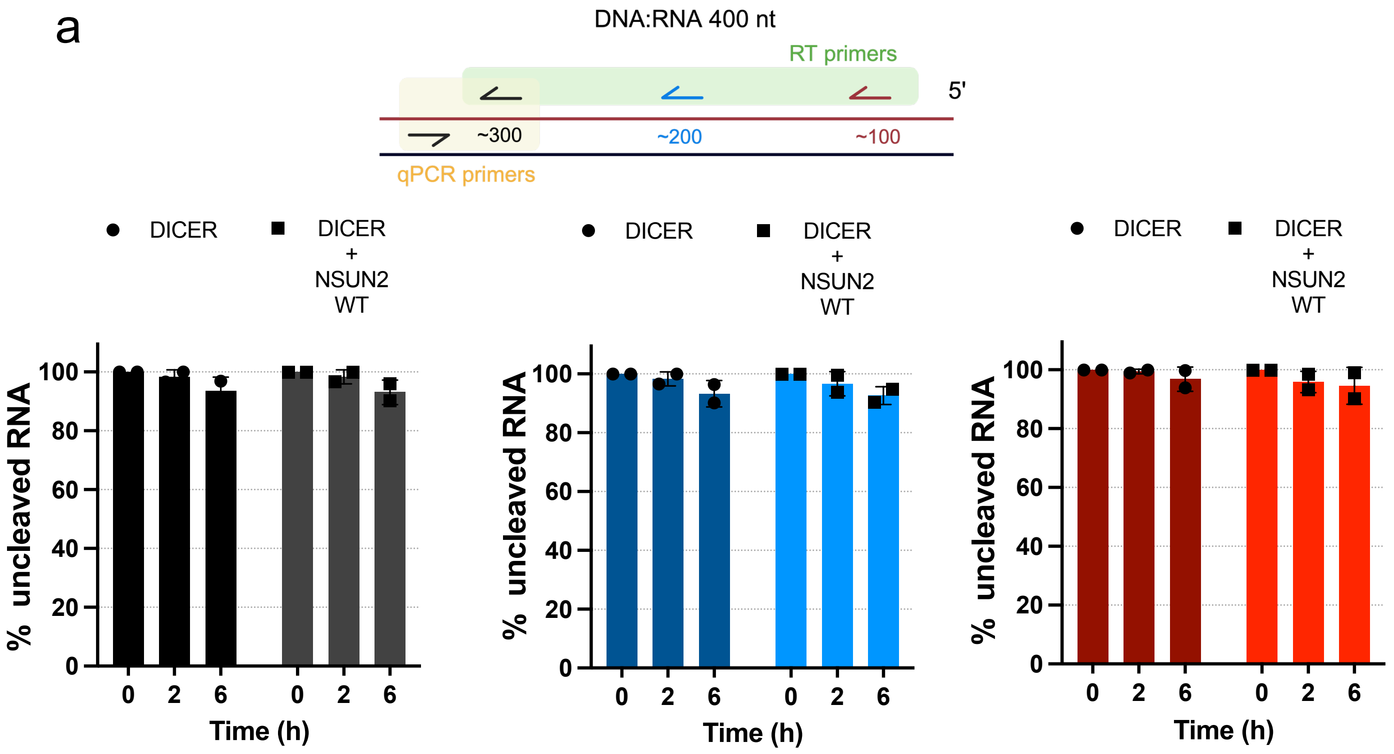

b

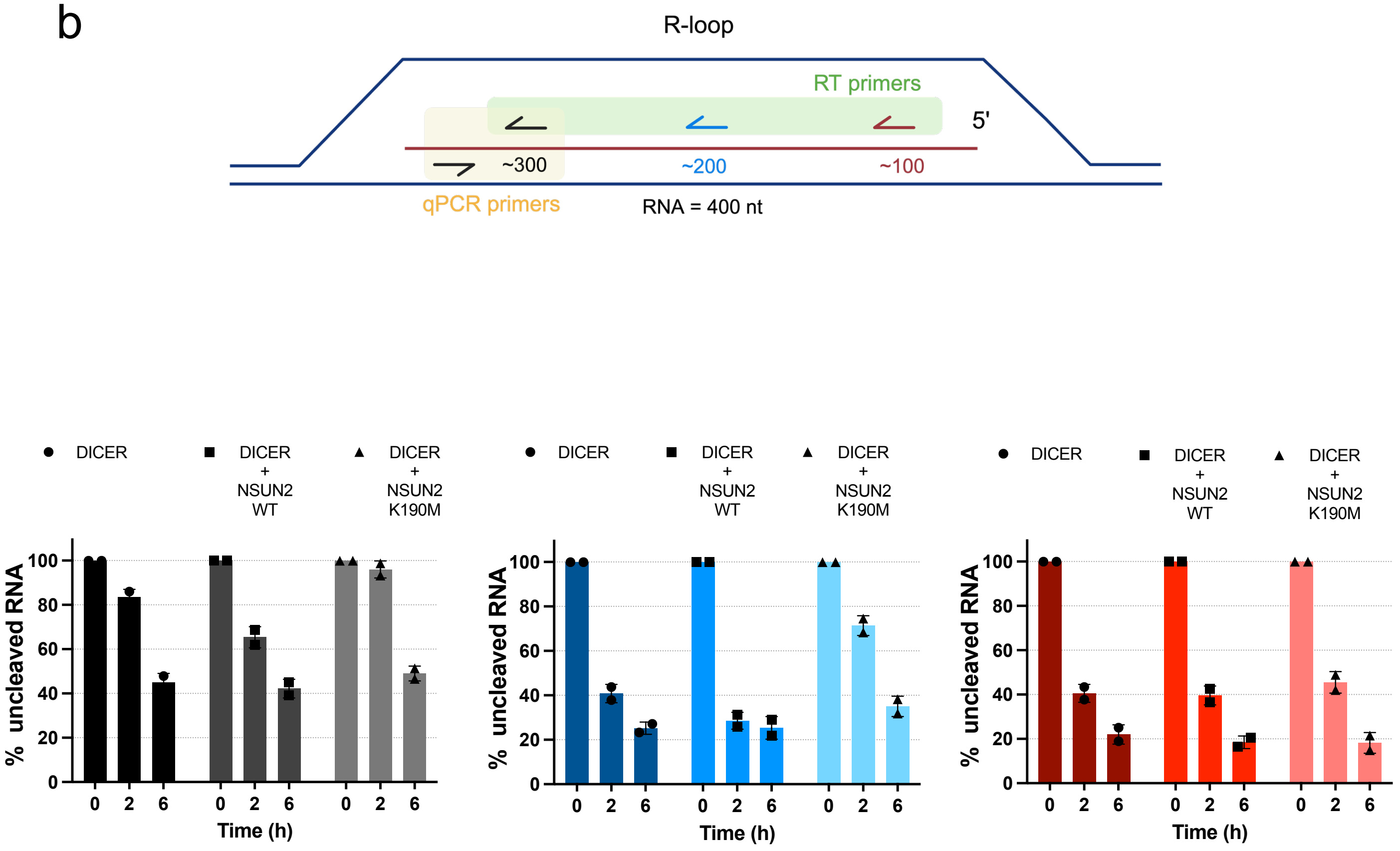

Supplementary Fig. 15

a

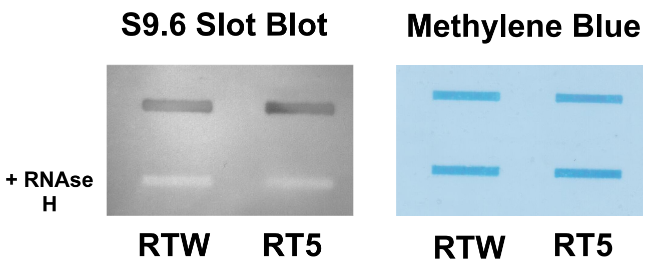

b

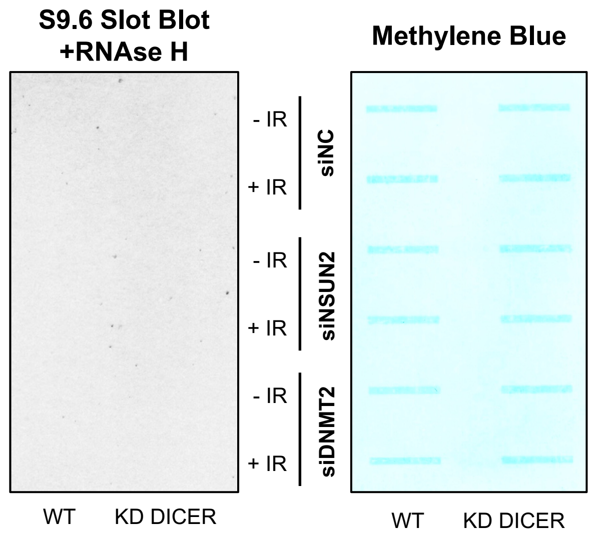

c

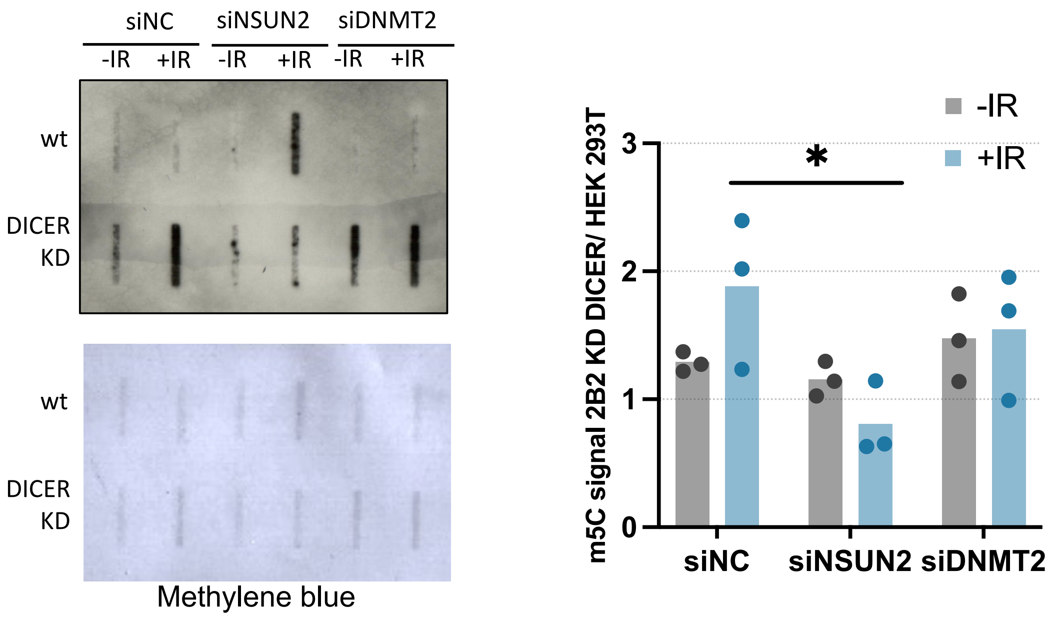

Supplementary Fig. 16

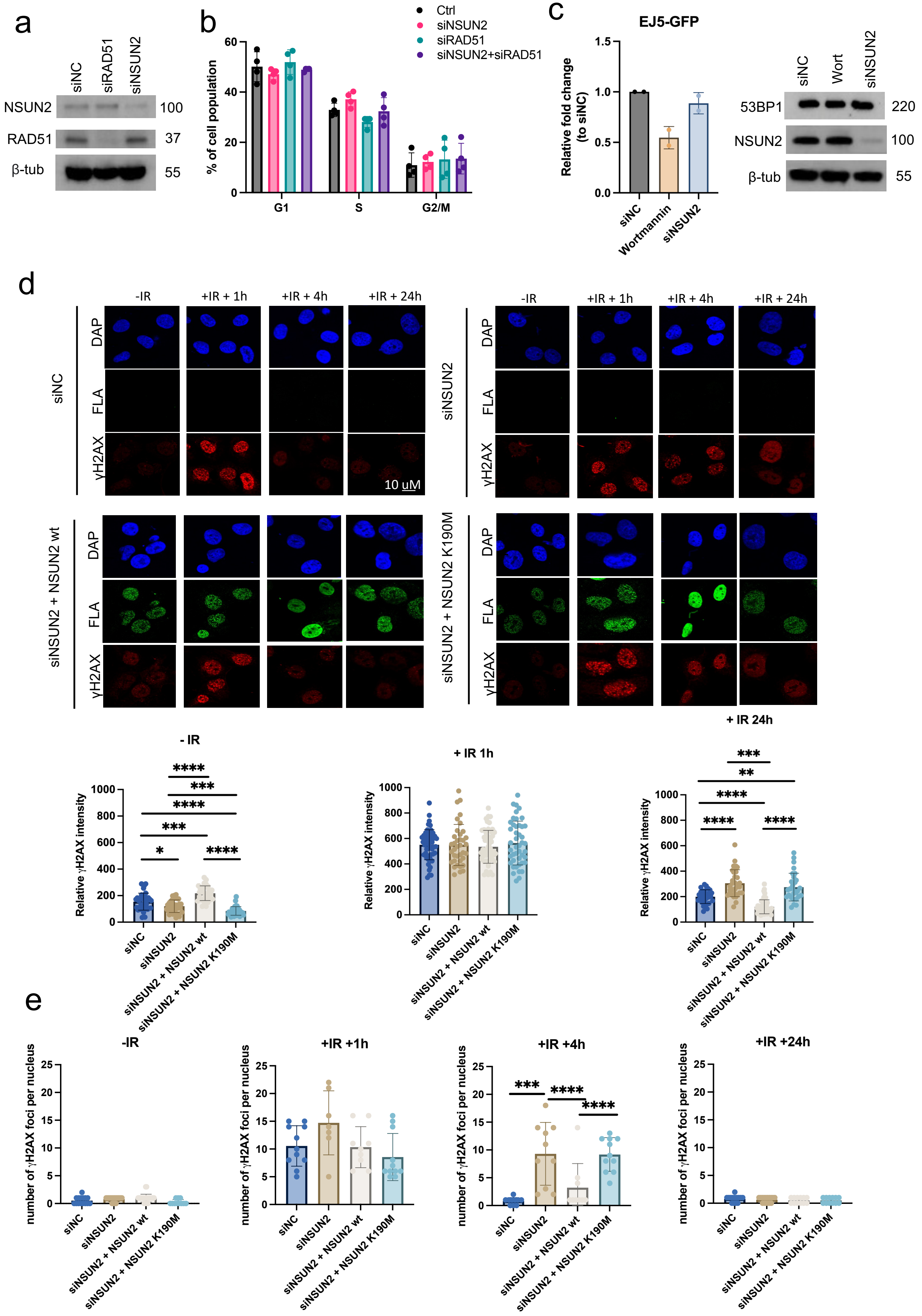

Supplementary Fig. 1

a) Left: representative proximity ligation assay (PLA) in combination with γH2AX immunofluorescence images showing the interaction between NSUN2 and γH2AX in U2OS cells treated with ionizing radiation (+IR) in dose dependent manner (0, 2, 5 and 10 Gy, 15 min). Cells were co-stained with γH2AX antibody (shown in green channel) and DAPI (blue channel). Scale bars, 10 μm.

Right: quantification of PLA nuclear foci. Data are presented as mean values ± Standard Deviation (SD). Significance is indicated as follows: **** $p \leq 0.0001$.

b) Left: representative proximity ligation assay (PLA) in combination with γH2AX immunofluorescence images showing the interaction between HOXD11 and γH2AX in U2OS cells treated with ionizing radiation (+IR, 10 Gy). Cells were co-stained with γH2AX antibody (shown in green channel) and DAPI (blue channel). Scale bars, 10 μm. Right: quantification of PLA nuclear foci. Non-significant comparisons ($p > 0.05$) are not shown.

c) Immunofluorescence images showing expression of HOXD11 (red channel) and γH2AX (green channel) in U2OS cells in no damage (-IR) and damage (+IR, 10 Gy, 15 min) conditions. Cells were co-stained with DAPI (blue channel). Scale bars, 10 μm.

d) Right: representative images showing the recruitment of NSUN2 to sites of laser-induced DNA damage in HEK293T cells transiently transfected with either NeonGreen-NSUN2 or NeonGreen-NSUN2 K190M plasmids. The irradiated regions of interest (ROIs) are marked by red dashed lines. Images were taken before and after laser microirradiation to visualize NSUN2 localization dynamics. DNA damage was induced using a 405 nm laser and Hoechst 33342 pre-sensitization. Cells were treated with or without Triptolide (TPL, 10 μM, 1 h) to inhibit transcription. Scale bars, 5 μm. Bottom left: quantification of relative NSUN2 enrichment at damage sites over time (0–132 seconds), expressed as the ROI fluorescence intensity relative to background (ROI/background). n=3. Data are presented as mean values ± Standard Error (SEM). Statistical analysis was performed using two-way ANOVA with multiple comparisons. Significance is indicated as follows: **** $p \leq 0.0001$.

Source data are provided as Source Data file.

Supplementary Fig. 2

a) PCA plot representing genome-wide signal variance from ChIP-seq datasets of NSUN2 and input samples in untreated (–4OHT) or treated with (Z)-4-hydroxytamoxifen (+4OHT). Each point represents one biological replicate.

b-f) Metagene plots and box plots displaying normalized ChIP-seq signal for NSUN2 (red) and input (blue) across a 2.5 kb window flanking annotated either uncut AsiSI restriction sites, HR and NHEJ-prone, highly transcriptionally active and low transcriptionally active AsiSI-induced DSB sites (centred at position 0).

g) snapshot depicting NSUN2 ChIP-seq signal (red) and corresponding input control (blue) at the VSTM2B gene locus in DIvA U2OS cells, either untreated (–4OHT) or treated with (Z)-4-hydroxytamoxifen (+4OHT). The VSTM2B locus is a representative transcriptionally active AsiSI site.

h) ChIP-qPCR validation of NSUN2 enrichment at the DS1 AsiSI (RBMXL1) site in DIvA U2OS cells. Top: Schematic representation of the DS1 AsiSI site, with the double-strand break location (red dashed line) and positions of qPCR primers indicated. Distances from the cut site are shown in nucleotides. Bottom: ChIP-qPCR analysis of NSUN2 enrichment at DS1, with GAPDH as a reference locus, in DIvA U2OS cells following (Z)-4-hydroxytamoxifen (+4OHT) treatment. Statistical significance was determined using the non-parametric Mann-Whitney test. Error bars represent mean ± SEM, n = 3 biological replicates. *$p \leq 0.05$.

Source data are provided as Source Data file.

Supplementary Fig. 3

a) Graphical summary of the chr-RNA isolation and Direct RNA Sequencing workflow. Created in BioRender.com (https://BioRender.com/plxknze). AsiSI-ER U2OS cells were treated with (Z)-4-hydroxytamoxifen (+4OHT) to induce site-specific DNA double-strand breaks (DSBs). Following treatment, nuclei were isolated and subjected to nuclear fractionation to separate chromatin-associated RNA (cheRNA) and soluble nucleoplasmic RNA. Chromatin-bound RNA fractions were then subjected to ribosomal RNA (rRNA) depletion using magnetic bead-based capture of rRNA species. The rRNA-depleted samples were used as input for Direct RNA Sequencing (DRS) via Oxford Nanopore Technology (ONT). The sequencing output was analysed through bioinformatic pipelines to identify and quantify RNA modifications, including m^5C. Created with BioRender.com. BioRender (https://BioRender.com/plxknze).

b) Metagene analysis of m^5C presence at all AsiSI cut sites. Line plot representing the distribution of m^5C-modified residues detected by DRS across a ±2 kb window flanking all annotated AsiSI-induced double-strand break (DSB) sites. Red dashed line indicates cells treated with (Z)-4-hydroxytamoxifen (+4OHT) and transfected with siNSUN2, while the solid

blue line represents control cells treated with +4OHT alone. Each point reflects the average m^5C modification frequency per position across all sites.

c) Boxplot comparing the distribution of 5-methylcytosine (m^5C) modification signal in chromatin-associated RNA in the absence of NSUN2 (siNSUN2 +4OHT, blue) and presence of NSUN2 (+4OHT, orange) upon DNA damage induced by (Z)-4-Hydroxytamoxifen (4OHT). m^5C signal was quantified within ±2.5 kb flanking annotated AsiSI cut sites using direct RNA sequencing (DRS). Statistical significance was assessed using a two-sided Wilcoxon test. n=3.

d) Metagene analysis of m^5C distribution at NSUN2 ChIP-seq peak regions upon DNA damage. Line plot represents the averaged m^5C modification frequency across ±2 kb windows centred on NSUN2 ChIP-seq peaks in +4OHT-treated wild-type cells (blue line) and NSUN2 knockdown cells (dashed red line, siNSUN2 +4OHT).

Supplementary Fig. 4

a) DRS nanopore sequencing analysis of m^5C RNA modification at the *PCGF1* locus upon ASiSI-induced DNA double-strand break (DSB) following 4-OHT treatment. The top track shows the genomic region on chromosome 2 (chr2:74,505,262–74,508,753; hg38), with the *PCGF1* gene annotated in blue. Red squares indicate the presence of m^5C RNA modifications across the locus. Data are shown for conditions with negative control siRNA (+4OHT siNC) and NSUN2 knockdown (+4OHT siNSUN2). The ASiSI cut site is annotated as a blue block in the lower track.

b) DRS nanopore sequencing analysis of m^5C RNA modification at the *HEBP1* locus upon ASiSI-induced DNA double-strand break (DSB) following +4-OHT treatment. The genomic region on chromosome 12 (chr12:13,000,869–13,003,709; hg38) is shown, with the *HEBP1* gene annotated in blue. m^5C RNA modifications are indicated by red squares, representing modification sites along individual reads. Two experimental conditions are displayed: +4OHT siNC (Negative Control) and +4OHT siNSUN2 (NSUN2 knockdown). The ASiSI restriction site is marked as a blue block.

c) Zoomed-in view of m^5C RNA modifications at the *HEBP1* locus. DRS nanopore sequencing data display a 121 bp region on chromosome 12 (chr12:13,001,271–13,001,390; hg38) upon ASiSI-induced DNA damage (+4OHT treatment). Red squares indicate individual m^5C modifications across RNA reads. The boxed region (in blue) highlights a cluster of NSUN2-dependent methylation sites, located approximately 300 bp downstream of the nearest ASiSI-induced double-strand break site. Genomic DNA sequence is shown at the bottom.

Supplementary Fig. 5

a-c) Left: representative images showing zC-FISH-PLA of NSUN2 and DNA probes (DS2 AS, DS2 SS and DS2 SCR) with (+4OHT) or without (-4OHT) and (Z)-4-Hydroxytamoxifen incubation for 4h in NSUN2 depleted (siNSUN2), siNegative Control (siNC). Right: quantification plot zC-FISH-PLA nuclear foci. Non-significant p-values are not displayed.

d) Left: representative images showing zC-FISH-PLA detecting interaction between NSUN2 and DNA probe (DS2 antisense strand) in the absence of 5-azacytidine (zC) pre-incubation. Right: quantification of nuclear FISH-PLA foci. Significance is indicated as follows: ****$p \leq 0.0001$.

Source data are provided as Source Data file.

Supplementary Fig. 6

a) Graphical schematic of the DS3 locus used for FISH-PLA targeting an intergenic AsiSI-induced DNA double-strand break (DSB). The DSB site is located within a non-coding region and, the FISH-PLA probe design includes eight DNA oligonucleotides positioned on the sense strand, beginning approximately 100 base pairs downstream of the break site and spaced at ~40 bp intervals. Created in BioRender (https://BioRender.com/gtzxlv3).

b) Left: zC-FISH-PLA analysis of NSUN2 interaction with DNA probes targeting the intergenic DS3 locus treated with (+4OHT) or without (−4OHT) (Z)-4-Hydroxytamoxifen. Right: quantification plot zC-FISH-PLA nuclear foci. Data are presented as mean values ± Standard Deviation (SD). n=3. Statistical significance was determined using the two-tailed non-parametric Mann-Whitney test. Non-significant p-values are not displayed.

c–d) Left: zC-FISH-PLA detection of DNMT2 in association with DNA probes targeting either the antisense (DS2 AS) or sense strand (DS2 SS) of the DS2 locus, in the presence (+4OHT) or absence (−4OHT) of (Z)-4-Hydroxytamoxifen. Right: quantification of nuclear FISH-PLA foci. Non-significant p-values are not displayed.

Source data are provided as Source Data file.

Supplementary Fig. 7

a) PCA plots show a comparison of chrRNA-Seq coverage in the 5kb flanking region of BLESS 80 *Asi*SI sites between sample replicates in NSUN2 knockdown and control conditions. n=3.

b) Heatmaps illustrate nascent RNA transcription (chrRNA-seq) signal intensity over ±5 kb windows flanking annotated AsiSI-induced DNA double-strand break (DSB) sites, ordered by increasing cleavage efficiency. Left panel: sense strand reads. Right panel: antisense strand reads. Conditions shown include NSUN2 knockdown (+4OHT, siNSUN2), control knockdown (+4OHT, siCtrl), and undamaged cells (-4OHT). Each row corresponds to a DSB site; colour intensity reflects normalized read coverage (see scale bars).

c) Metagene plot shows chrRNA-Seq sense and antisense coverage in wt (siCtrl) and NSUN2 knockdown cells with damage induction (+4-OHT) around 2.55kb flank region of Uncut AsiSI sites.

d) Representative snapshot of the SRSF6 gene locus showing sense (red) and antisense (blue) chromatin-associated RNA sequencing (chrRNA-Seq) signal upon (Z)-4-Hydroxytamoxifen (+4OHT) treatment in NSUN2 knockdown (siNSUN2) and wt (siCtrl) conditions. The displayed region spans ~2.5 kb flanking the AsiSI-induced double-strand break site

Supplementary Fig. 8

a) Box plot showing the distribution of $\log_2$ fold change of chrRNA-Seq coverage of sense reads and antisense reads upon NSUN2 knockdown in damage conditions compared to control for BLESS 80 AsiSI cut sites (+/- 500bp).

b) Representative immunofluorescence images showing γH2AX staining (red) in U2OS AsiSI-ER cells treated with (Z)-4-Hydroxytamoxifen (+4OHT, 4 h) in control (siNC) or NSUN2-depleted (siNSUN2) conditions. DAPI (blue) marks the nuclei. Right panel: quantification of γH2AX nuclear foci per nucleus. Significance is indicated as follows: ****$p \leq 0.0001$.

c) Box plots show the distribution of $\log_2$ fold change of chrRNA-Seq coverage of sense reads and antisense reads upon NSUN2 knockdown in damage conditions compared to control for AsiSI associated with highly transcribed regions (+/- 500bp).

d) Metagene plot shows chrRNA-Seq sense and antisense coverage in control (siCtrl) and NSUN2 knockdown cells with damage induction (+4-OHT) around 2.5kb flank region of AsiSI sites associated with low transcription.

e-f) Box plots showing the distribution of $\log_2$ fold change of chrRNA-Seq coverage of sense reads and antisense reads upon NSUN2 knockdown in damage conditions compared to control for AsiSI associated with low transcription and HR (+/- 500bp).

g) Metagene plot shows chrRNA-Seq sense and antisense coverage in control (siCtrl) and NSUN2 knockdown cells with damage induction (+4-OHT) around 2.5kb flank region of NHEJ prone AsiSI sites.

h) Box plots show the distribution of log_2 fold change of chrRNA-Seq coverage of sense reads and antisense reads upon NSUN2 knockdown in damage conditions compared to control for NHEJ prone AsiSI (+/- 500bp).

Supplementary Fig. 9

a) Coomassie Brilliant Blue staining of NSUN2 and NSUN2 K190M protein purification.

b) Coomassie Brilliant Blue staining of DICER and DICER DEDE (D1320A; E1444A; D1709A; E1813A) protein purification.

c) Coomassie Brilliant Blue staining showing purified DICER protein following concentration using a 100 kDa molecular weight cut-off spin column.

Supplementary Fig. 10

a) Proximity Ligation Assay (PLA) of NSUN2 and DICER under conditions with (+IR, 10 Gy, 15 min) or without (–IR) ionizing radiation including NSUN2 knockdown condition, post-fixation treatment with RNases A, T1, and III (AT3) and, transcriptional inhibition with either 5,6-dichloro-1-β-D-ribofuranosylbenzimidazole (DRB) or triptolide (TPL).

b) Left: representative immunofluorescence images of proximity ligation assay (PLA) targeting 53BP1 and γH2AX in U2OS cells. Experimental conditions include untreated cells (-IR), ionizing radiation-treated cells (+IR, 10 Gy, 15 min), and cells treated post-fixation with a cocktail of RNases A, T1, and III (AT3) following IR. Nuclei are counterstained with DAPI (blue); PLA signal appears as red foci indicating the spatial proximity of 53BP1 and γH2AX. Right: quantification of PLA nuclear foci per nucleus. Significance is indicated as follows: ****$p \leq 0.0001$.

c) Proximity ligation assay (PLA) detecting DNMT2 and DICER interaction in U2OS cells with or without exposure to ionizing radiation (IR, 10 Gy, 15 min). A single-antibody condition using DNMT2 alone serves as a negative control. Left: Representative fluorescence images showing PLA signal (red) and nuclei stained with DAPI (blue). Right: quantification of nuclear PLA foci per condition. Non-significant p-values are not displayed.

Source data are provided as Source Data file.

Supplementary Fig. 11

a-d) Representative images of FISH-PLA targeting DICER in combination with either DS2 antisense (AS) or scrambled (SCR) DNA probes, in U2OS cells treated with or without (Z)-4-

Hydroxytamoxifen (+/−4OHT). Experimental conditions include NSUN2 knockdown (siNSUN2), DICER knockdown (siDICER), and post-fixation treatment with RNase H.
e) Representative single plane confocal microscopy images of FISH-PLA detecting DICER in proximity to the DS2 antisense DNA probe (red) in DIvA U2OS cells. DNA damage foci are visualized by γH2AX immunofluorescence (green), and nuclei are counterstained with DAPI (blue). Merged images show colocalization of PLA signals with DNA damage sites. White boxes indicate regions selected for zoom-in views shown on the right, highlighting the spatial overlap between PLA signals and γH2AX foci. Scale bar: 10 µm. Zoom-in panels: scale bar 3 µm.
Source data are provided as Source Data file.

Supplementary Fig. 12
a) Northern blot of DICER cleavage reaction and in vitro transcribed (IVT) ssRNA DS2 derived after incubation with NSUN2 wt only, DICER wt and NSUN2, DICER DEDE and NSUN2 wt, DICER wt and NSUN2 K190M at certain time points (0, 2h, 6h). ssRNA Marker (Low Range ssRNA Ladder, NEB # N0364S). Quantification plot of cleaved ssRNA along the time points considered. Errors bars, mean ± SD. n=3.
b) Northern blot of DICER cleavage reaction and IVT dsRNA and DNA:RNA heteroduplex DS2 derived after incubation with NSUN2 wt only, DICER wt and NSUN2, DICER DEDE and NSUN2 wt, DICER wt and NSUN2 K190M at certain time points (0, 2h, 6h). Quantification plot of cleaved dsRNA along the time points considered. Errors bars, mean ± SD. n=3.
Source data are provided as Source Data file.

Supplementary Fig. 13
a) Top: Schematic representation of in vitro transcribed (IVT) antisense single stranded RNA DS2 derived with RT primers: (red arrow ~100 nt, blue arrow ~200 nt and black arrow ~300nt away from the *Asi*Si site, highlighted in green), and qPCR primers (black arrows, highlighted in yellow). Created in BioRender (https://BioRender.com/umosg77).
Bottom: quantification plots of cleaved RNA at 0, 2h and 6h time points in presence of DICER only, DICER + NSUN2 wt and DICER + NSUN2 K190M. Black, blue and red bars correspond to black, blue and red primer specific RT, respectively. Errors bars, mean ± SD. n=2.

b) Top: Schematic representation of IVT double stranded RNA DS2 derived with RT primers: (red arrow ~100 nt, blue arrow ~200 nt and black arrow ~300nt away from the *Asi*Si site, highlighted in green), and qPCR primers (black arrows, highlighted in yellow). Created in BioRender (https://BioRender.com/t6be2y0).
Bottom: quantification plots of cleaved RNA at 0, 2h and 6h time points in presence of DICER only, DICER + NSUN2 wt and DICER + NSUN2 K190M. Black, blue and red bars correspond to black, blue and red primer specific RT, respectively. Errors bars, mean ± SD. n=2.
Source data are provided as Source Data file.

Supplementary Fig. 14
a) Top: Schematic representation of DNA:RNA heteroduplex DS2 derived with RT primers: (red arrow ~100 nt, blue arrow ~200 nt and black arrow ~300nt away from the *Asi*Si site, highlighted in green), and qPCR primers (black arrows, highlighted in yellow). Created in BioRender (https://BioRender.com/2wt0za3).
Bottom: quantification plots of cleaved RNA at 0, 2h and 6h time points in presence of DICER only and DICER + NSUN2 wt. Black, blue and red bars correspond to black, blue and red primer specific RT, respectively. Errors bars, mean ± SD. n=2.
b) Top: Schematic representation of *in vitro* transcribed (IVT) DS2 antisense RNA forming R-loop with RT primers: (red arrow ~100 nt, blue arrow ~200 nt and black arrow ~300nt away from the *Asi*Si site, highlighted in green), and qPCR primers (black arrows, highlighted in yellow). Created in BioRender (https://BioRender.com/m14bqlt).
Bottom: quantification plots of cleaved RNA at 0, 2h and 6h time points in presence of DICER only, DICER + NSUN2 wt and DICER + NSUN2 K190M. Black, blue and red bars correspond to black, blue and red primer specific RT, respectively. Errors bars, mean ± SD. n=2.
Source data are provided as Source Data file.

Supplementary Fig. 15
a) Left: slot blot analysis using the S9.6 antibody on R-loop 5′-tail substrates containing either unmodified RNA (RTW) or m^5C-modified RNA (RT5). Samples were treated with RNase H to confirm r-loop specificity. Right: methylene blue staining as a loading control.
b) Left: slot blot showing S9.6 signal from genomic DNA samples treated with RNase H. Right: methylene blue staining as a loading control.

c) Top: Slot Blot probing with m^5C antibody on nuclear RNA isolated from cells treated with (+IR, 10 Gy, 10 min) or without (-IR) Ionizing Radiation, in NSUN2 depleted (siNSUN2), DNMT2 depleted (siDNMT2), siNegative Control (siNC), and HEK 293T or HEK 2B2 depleted for DICER protein. Bottom: methylene blue blot used as a loading control. Right: quantification of the slot blots. Significance is indicated as follows: $*p \leq 0.05$.
Source data are provided as Source Data file.

Supplementary Fig. 16

a) Western Blot showing the NSUN2 and RAD51 knockdown efficiency related to MTS assay.
b) Bar chart for cell cycle distribution in wt condition (Ctrl) in absence of NSUN2 (siNSUN2), in knockdown RAD51 cells (siRAD51) and in double depleted (siNUN2 + siRAD51) condition. Statistical significance was determined using the two-way ANOVA test. Non-significant p-values are not displayed.
c) Left: efficiency of NHEJ repair in EJ5-GFP reporter system in NSUN2 depleted (siNSUN2), siNegative Control (siNC) conditions and after incubation with Wortmannin. Errors bars, mean ± SD. n=2. Right: Western Blot of the 53BP1 and NSUN2 knockdown efficiency related to EJ5-GFP reporter assay.
d) Top: representative immunofluorescence images of FLAG-tagged NSUN2 wt, FLAG-tagged NSUN2 K190M and γH2Ax in NSUN2 depleted (siNSUN2), siNegative Control (siNC), NSUN2 wt rescued (siNSUN2 + NSUN2 wt) and NSUN2 K190M rescued (siNSUN2 + NSUN2 K190M) irradiated with (+IR +1h; +IR + 4h; +IR +24h) or without (-IR) 5 Gy. Bottom: Quantification plot of the γH2Ax intensity. Significance is indicated as follows: $*p \leq 0.05$, $**p \leq 0.01$, $***p \leq 0.001$, $****p \leq 0.0001$. Non-significant p-values are not displayed.
e) Quantification of γH2Ax foci per nucleus from d). Significance is indicated as follows: $***p \leq 0.001$, $****p \leq 0.0001$. Non-significant p-values are not displayed.
Source data are provided as Source Data file.
