# Supplementary material for: NSUN2 facilitates DICER cleavage of DNA damage-associated R-loops to promote repair
Source: Nat Commun. 2025 Aug 23;16:7882. doi: 10.1038/s41467-025-63220-9 (PMC12374970; doi:10.1038/s41467-025-63220-9)
Supplement: Supplementary file 2 — Description of Additional Supplementary Files [file 41467_2025_63220_MOESM2_ESM.pdf]

Description of Additional Supplementary Files

Supplementary Data 1: List of Primers, antibodies and plasmids used in this study.
