# Supplementary material for: NSUN2 facilitates DICER cleavage of DNA damage-associated R-loops to promote repair
Source: Nat Commun. 2025 Aug 23;16:7882. doi: 10.1038/s41467-025-63220-9 (PMC12374970; doi:10.1038/s41467-025-63220-9)
Supplement: Supplementary file 4 — Reporting Summary [file 41467_2025_63220_MOESM4_ESM.pdf]

nature portfolio

Corresponding author(s): Monika Gullerova
Last updated by author(s): 09/06/2025

Reporting Summary

Nature Portfolio wishes to improve the reproducibility of the work that we publish. This form provides structure for consistency and transparency in reporting. For further information on Nature Portfolio policies, see our Editorial Policies and the Editorial Policy Checklist.

Statistics

For all statistical analyses, confirm that the following items are present in the figure legend, table legend, main text, or Methods section.

n/a	Confirmed	
☐	☒	The exact sample size (*n*) for each experimental group/condition, given as a discrete number and unit of measurement
☒	☐	A statement on whether measurements were taken from distinct samples or whether the same sample was measured repeatedly
☐	☒	The statistical test(s) used AND whether they are one- or two-sided<br>*Only common tests should be described solely by name; describe more complex techniques in the Methods section.*
☒	☐	A description of all covariates tested
☒	☐	A description of any assumptions or corrections, such as tests of normality and adjustment for multiple comparisons
☐	☒	A full description of the statistical parameters including central tendency (e.g. means) or other basic estimates (e.g. regression coefficient) AND variation (e.g. standard deviation) or associated estimates of uncertainty (e.g. confidence intervals)
☐	☒	For null hypothesis testing, the test statistic (e.g. *F*, *t*, *r*) with confidence intervals, effect sizes, degrees of freedom and *P* value noted<br>*Give P values as exact values whenever suitable.*
☒	☐	For Bayesian analysis, information on the choice of priors and Markov chain Monte Carlo settings
☒	☐	For hierarchical and complex designs, identification of the appropriate level for tests and full reporting of outcomes
☒	☐	Estimates of effect sizes (e.g. Cohen's *d*, Pearson's *r*), indicating how they were calculated

Our web collection on statistics for biologists contains articles on many of the points above.

Software and code

Policy information about availability of computer code

Data collection

Illumina sequencing

Data analysis

ChromRNASeq Data Processing
Adapters from ChromRNASeq libraries were trimmed using Cutadapt (version 4.4) (https://cutadapt.readthedocs.io/en/stable/installation.html) in paired-end mode. The quality of the resulting FASTQ files was assessed using FastQC (https://www.bioinformatics.babraham.ac.uk/projects/fastqc/). Trimmed reads were aligned to the human hg19 reference genome using STAR aligner. BAM files were then strand-separated into positive and negative alignments using Samtools (https://www.htslib.org/).
Metagene Plots
Strand-specific coverage files representing CPM-normalized read counts per nucleotide were generated using the bamCoverage function from deepTools (https://deeptools.readthedocs.io/en/develop/). The computeMatrix function was then used on strand-separated bigWig files to compute CPM coverage in the ±2.5 kb flanking regions around AsiSI sites, with a bin size of 1 nucleotide. bedtools intersect was used to identify gene regions overlapping these flanks. A custom Python script annotated each bin in the positive strand matrix as either sense or antisense, based on the orientation of nearby genes. The same logic was applied to the negative strand matrix. Only bins overlapping genes were used for annotation.
Sense bins from both positive and negative strand matrices were concatenated to form a unified sense matrix. Antisense matrices were constructed similarly. For each AsiSI site, read counts from overlapping genes were summed so that each row represented one site and each column one bin (total of 5000 bins for ±2.5 kb). Matrices were categorized based on site annotations provided by Prof. Gaëlle Legube (e.g., HR-prone, NHEJ-prone, uncut, transcriptionally active or inactive). Line plots and fill plots were generated using the matplotlib Python package.
PCA Plots
Read coverage within 5 kb of 80 AsiSI sites (BLESS-defined, courtesy of Prof. Gaëlle Legube) was summarized using bigwigSummary from

deepTools and compared across replicates using the plotPCA function.
Box Plots
CPM coverage values from sense and antisense matrices were summed across 500 bp flanking regions of each AsiSI site and visualized as box plots using matplotlib. Significance was tested using the two-sample Wilcoxon test from scipy. HR versus NHEJ coverage was compared using the Mann–Whitney U test.
Log2 fold changes were calculated as the ratio of CPM coverage in condition vs. control (for sense and antisense matrices separately). These were visualized as box plots and statistically assessed using the Wilcoxon test.
Heatmaps
The plotHeatmap function from deepTools was used to generate heatmaps centered on all annotated DSBs sorted by cleavage efficiency. Heatmaps were created separately for sense and antisense matrices.
Cumulative Distribution Plots
Cumulative distribution plots showing fold changes in read coverage (±500 bp around AsiSI sites) between condition and control were generated using a custom Python script. Statistical significance between sense and antisense distributions was evaluated using the two-sample Wilcoxon test from scipy.
BLESS-Seq Data Processing
BLESS-Seq data (E-MTAB-5817) were processed as previously described. Read coverage within ±500 bp of DSBs was computed using bedtools multicov, and sites were ranked based on read count to estimate cleavage efficiency.
ChIP-Seq Data Processing
ChIP-Seq libraries were sequenced using Illumina NovaSeq X Plus 500 (paired-end, 150 bp). FastQC was used to assess read quality before and after trimming. Reads were aligned to the hg19 genome using BWA (http://bio-bwa.sourceforge.net/). Duplicate reads were removed, and alignments were sorted and indexed using Samtools (https://www.htslib.org/). bamCompare from deepTools was used to calculate log2 fold change (+4OHT vs. −4OHT). Peaks were called using MACS2 (https://github.com/macs3-project/MACS) with a q-value cutoff of 0.01 and required to be present in all three replicates.
ChIP-Seq PCA Plot
Coverage around ±2.5 kb of AsiSI sites was quantified using bedtools multicov, normalized to CPM, and analyzed using the PCA function from scikit-learn.
ChIP-Seq Metagene Plot
The computeMatrix function from deepTools was used to generate average profiles of log2 fold changes (+4OHT/−4OHT) in ±2.5 kb regions around DSBs. Bin size was set to 1.
ChIP-Seq Box Plot
Read coverage in ±2.5 kb regions of DSBs were quantified with bedtools multicov and normalized to CPM. Box plots were created in matplotlib, and the Wilcoxon test was used to determine significance ($P < 0.01$) using scipy.
ChIP-Seq IGV Profiles and Heatmaps
CPM-normalized bigWig files were generated using bamCoverage and visualized in IGV (https://software.broadinstitute.org/software/igv/). Heatmaps of all annotated DSBs were generated using plotHeatmap from deepTools.
ONT Data Processing
Oxford Nanopore RNA004 reads were basecalled using Dorado (https://github.com/nanoporetech/dorado) with HAC and RNA modification models for Ψ, m5C, m6A, and inosine. Reads were aligned to the hg38 genome using the Dorado aligner with parameters “-ax splice -k 14”. Modified bases were identified using modkit pileup (https://github.com/nanoporetech/modkit) with a modification threshold of 0.7. Output bedMethyl tables were used for all downstream analysis.
ONT Box Plots
Differential m5C modification between conditions was assessed using modkit dmr pair. Regions of interest included ±2.5 kb flanks of AsiSI sites and NSUN2 ChIP-Seq peaks. Counts of modified residues were visualized as box plots in matplotlib and statistically tested using the Wilcoxon test.
ONT m5C Metagene Plot
The modkit localize command was used to extract the percentage of m5C-modified residues at each position within ±2 kb around AsiSI sites and NSUN2 ChIP-Seq peak summits. Results were plotted using matplotlib.
ONT Snapshots
Aligned ONT reads were visualized using IGV. Reads were grouped by strand, and m5C modification probabilities were shown using the “show modification” option.

For manuscripts utilizing custom algorithms or software that are central to the research but not yet described in published literature, software must be made available to editors and reviewers. We strongly encourage code deposition in a community repository (e.g. GitHub). See the Nature Portfolio guidelines for submitting code & software for further information.

Data

Policy information about availability of data

All manuscripts must include a data availability statement. This statement should provide the following information, where applicable:

- Accession codes, unique identifiers, or web links for publicly available datasets
- A description of any restrictions on data availability
- For clinical datasets or third party data, please ensure that the statement adheres to our policy

Data reported in this paper can be shared by the lead contact upon request.
ChrRNA-seq data have been deposited to GEO and can be accessed under GSE260748 with private token: arubcusqrhabhit and GSE246729.
NSUN2 ChIP-seq data have been deposited to GEO and can be accessed under GSE294470 with private token: alifcyeibjshrod.
Oxford Nanopore data are available from the lead contact upon request.
Any additional information required to re-analyse the data reported in this work paper is available from the lead contact upon request.

Research involving human participants, their data, or biological material

Policy information about studies with human participants or human data. See also policy information about sex, gender (identity/presentation), and sexual orientation and race, ethnicity and racism.

Reporting on sex and gender	n/a
Reporting on race, ethnicity, or other socially relevant groupings	n/a
Population characteristics	n/a
Recruitment	n/a
Ethics oversight	n/a

Note that full information on the approval of the study protocol must also be provided in the manuscript.

Field-specific reporting

Please select the one below that is the best fit for your research. If you are not sure, read the appropriate sections before making your selection.

☒ Life sciences ☐ Behavioural & social sciences ☐ Ecological, evolutionary & environmental sciences

For a reference copy of the document with all sections, see nature.com/documents/nr-reporting-summary-flat.pdf

Life sciences study design

All studies must disclose on these points even when the disclosure is negative.

Sample size	chrRNA-seq and ChIP-seq 3 biological replicates per conditions; qRT-PCR and ChIP analyses: 3 biological replicates per condition. It is standard in molecular biology to use at least 3 biological replicates for experimental analyses.
Data exclusions	No data was excluded.
Replication	All attempts at replication were successful.
Randomization	This is not relevant to our study as we normally control for experimental samples by other methods.
Blinding	n/a

Reporting for specific materials, systems and methods

We require information from authors about some types of materials, experimental systems and methods used in many studies. Here, indicate whether each material, system or method listed is relevant to your study. If you are not sure if a list item applies to your research, read the appropriate section before selecting a response.

Materials & experimental systems

n/a	Involved in the study	
☐	☒	Antibodies
☐	☒	Eukaryotic cell lines
☒	☐	Palaeontology and archaeology
☒	☐	Animals and other organisms
☒	☐	Clinical data
☒	☐	Dual use research of concern
☒	☐	Plants

Methods

n/a	Involved in the study	
☒	☐	ChIP-seq
☒	☐	Flow cytometry
☒	☐	MRI-based neuroimaging

Antibodies

Antibodies used	NSUN2 Rabbit Polyclonal Proteintech #20854-1-AP<br>NSUN2 Mouse Monoclonal Proteintech #66580-1-Ig<br>DICER [13D6] Mouse monoclonal Abcam #ab14601

DNMT2 [D-9] Mouse monoclonal Santa Cruz #sc-365001
BRCA1 Antibody (D-9) Mouse monoclonal Santa Cruz #sc-6954
RAD51 (F-11) Mouse monoclonal Santa Cruz #sc-398587
Beta-tubulin Rabbit polyclonal Abcam #ab6046
FLAG [M2] Mouse monoclonal Sigma-Aldrich #F1804
Phospho-gamma-H2AX (Ser139) Rabbit Monoclonal ThermoFisher #MA5-33062
S9.6 Mouse Monoclonal Sigma-Aldrich #ZMS1017
5-Methylcytosine Rabbit Monoclonal Antibody (RM231) ThermoFisher #MA5-24694

Validation

NSUN2 Rabbit Polyclonal Proteintech #20854-1-AP (from manufacturer's website: Calculated Molecular Weight 767 aa, 86 kDa. Observed Molecular Weight 100 kDa. IP : 0.5-4.0 ug for 1.0-3.0 mg of total protein lysate. WB : 1:5000-1:50000. IF : 1:750-1:3000.The mouse cytosine-5 RNA methyltransferase NSun2 is a component of the chromatoid body and required for testis differentiation. Hussain et al. Mol Cell Biol. 2013. KD Validated: Activity-based RNA-modifying enzyme probing reveals DUS3L-mediated dihydrouridylation. Wei Dai et al. Nat Chem Biol 2021).
NSUN2 Mouse Monoclonal Proteintech #66580-1-Ig (from manufacturer's website: Calculated Molecular Weight 86 kDa Observed Molecular Weight 100 kDa. WB : 1:5000-1:50000. IP : 0.5-4.0 ug for 1.0-3.0 mg of total protein lysate. IHC : 1:1000-1:4000. IF : 1:50-1:500. N6-methyladenosine modification of circNSUN2 facilitates cytoplasmic export and stabilizes HMGA2 to promote colorectal liver metastasis. Ri-Xin Chen et al. Nat Commun. 2019).
DICER [13D6] Mouse monoclonal Abcam #ab14601 (from manufacturer's website: ko validated, WB: 1:100-1-2000, band observed at 240 kDa, ChIP: use at an assay dependent concentration. Transcriptional suppression of Dicer by HOXB-AS3/EZH2 complex dictates sorafenib resistance and cancer stemness. Tseng CF et al.Cancer Sci.2022).
DNMT2 [D-9] Mouse monoclonal Santa Cruz #sc-365001 (from manufacturer's website: Calculated Molecular Weight 45 kDa. WB: 1:100-1:1000, IF: 1:50-1:500. Position 34 of tRNA is a discriminative element for m5C38 modification by human DNMT2. Huang, ZX. et al. Nucleic Acids Res.2021. m5C modification of mRNA serves a DNA damage code to promote homologous recombination. Hao Chen et al. Nat Commun. 2020).
BRCA1 Antibody (D-9) Mouse monoclonal Santa Cruz #sc-6954 (from manufacturer's website: Molecular Weight of BRCA1: 220 kDa. WB 1:100-1:1000, IF 1:50-500). Improving the sensitivity of in vivo CRISPR off-target detection with DISCOVER-Seq. Zou, RS. et al. Nat Methods. 2023).
RAD51 (F-11) Mouse monoclonal Santa Cruz #sc-398587 (from manufacturer's website: Molecular Weight of Rad51: 37 kDa.WB 1:100-1:1000,IF 1:50-1:500). RHOJ controls EMT-associated resistance to chemotherapy. Debaugnies, M. et al. Nature. 2023).
Beta-tubulin Rabbit polyclonal Abcam #ab6046 (from manufacturer's website: WB: 1/500. Detects a band of approximately 50 kDa (predicted molecular weight: 50 kDa). An RNA-targeting CRISPR-Cas13d system alleviates disease-related phenotypes in Huntington's disease models. Morelli et al. Nat Neurosci 2023).
FLAG [M2] Mouse monoclonal Sigma-Aldrich #F1804 (from manufacturer's website: IF 1:500. Roquin binds microRNA-146a and Argonaute2 to regulate microRNA homeostasis. Srivastava et al. Nat. commun 2015).
Phospho-gamma-H2AX (Ser139) Rabbit Monoclonal ThermoFisher #MA5-33062 (from manufacturer's website: WB and IF 0.5-2 µg/mL. p53 drives a transcriptional program that elicits a non-cell-autonomous response and alters cell state in vivo. Moyer et al. Proc Natl Acad Sci U S A. 2020).
S9.6 Mouse Monoclonal Sigma-Aldrich #ZMS1017 (from manufacturer's website: IF 1:1000. DNA-RNA heteropolymer duplex prepared by transcription of phi X174 single-stranded DNA with DNA-dependent RNA polymerase. Boguslawski, et al. J. Immunol Methods. 1986.
5-Methylcytosine Rabbit Monoclonal Antibody (RM231) ThermoFisher #MA5-24694 (from manufacturer's website: Dot blot: 0.5-2 µg/mL.The distribution pattern of 5-methylcytosine in rye chromosomes Kalinka et al. PLoS One. 2020).
The HOXD11 polyclonal antibody (Cat. No. 18734-1-AP, Proteintech) has been validated for specificity and performance across multiple applications and species. Western blotting shows a single band at the expected molecular weight (~35 kDa) in human, mouse, and rat lysates, confirming specificity. Immunohistochemistry and immunofluorescence demonstrate nuclear localization consistent with HOXD11's known function as a transcription factor. The antibody has been successfully used in peer-reviewed studies, including Cell Stem Cell (Low et al., 2019) for kidney organoid characterization, Cell Reports (Mu et al., 2024) in chromatin regulation studies, and Molecular Biotechnology (Peng et al., 2024) in laryngeal carcinoma, further supporting its reproducibility and reliability. Validation was performed using antigen-affinity purified antibody, and the results are consistent across different tissue types and experimental conditions.

The H2AX pS139 REAfinity™ antibody (Miltenyi Biotec) is a recombinant humanIgG1 monoclonal (clone REA502) validated for specific, sensitive detection of γ-H2AX (phospho Ser139) in both human and mouse samples. It has been confirmed to perform reliably in immunofluorescence and immunohistochemistry on formaldehyde-fixed cells (Miltenyi QC data), as well as in intracellular flow cytometry and MACSima Imaging Cyclic Staining (MICS) with recommended dilution at 1:50 using standard permeabilization protocols. Although Miltenyi's own documentation doesn't include Western blot data, similar reagents (BD Pharmingen's mouse anti γH2AX pS139) clearly detect a single ~15kDa band and show UV or etoposide-induced nuclear foci formation in HeLa and PBMCs, confirming assay specificity and DNA damage responsiveness . Overall, this antibody demonstrates robust specificity (nuclear foci), reproducibility (multiple modalities), and appropriate sensitivity for γ-H2AX detection in human/murine systems.

Eukaryotic cell lines

Policy information about cell lines and Sex and Gender in Research

Cell line source(s)	HeLa, HeLa EJ5-GFP, HeLa DR-GFP, HEK 293T, Oxford Cell and Tissue Biobank<br>HEK 293T 2B2 (gift from the Filipowicz's Lab),<br>U2OS and AsiSI-ER U2OS (gift from Legube's Lab)
Authentication	We have authenticated the knockdown of specific proteins by western blot.
Mycoplasma contamination	We can confirm that the cell lines were checked for mycoplasma contamination.

Commonly misidentified lines<br>(See ICLAC register)	n/a

Plants

Seed stocks	n/a
Novel plant genotypes	n/a
Authentication	n/a
